# Supplementary material for: De novo assembly and characterization of a highly degenerated ZW sex chromosome in the fish Megaleporinus macrocephalus
Source: Gigascience. 2024 Nov 26;13:giae085. doi: 10.1093/gigascience/giae085 (PMC11590113; doi:10.1093/gigascience/giae085)
Supplement: giae085_supplement_Files [file giae085_supplement_files.zip › supplementary_material_figures.pdf]

Summary

Supplementary Figure 1. Interspersed repeat landscape of *Megaleporinus macrocephalus* genome. **2**

Supplementary Figure 2. Gene Ontology (G.O) Terms of Cellular Component, Molecular Function and Biological Process domains of *Megaleporinus macrocephalus* genome. **3**

Supplementary Figure 3. The average number of markers in linkage groups (left y axis) and the number of linkage groups (right y axis) according to LOD score (x axis). **4**

Supplementary Figure 4. Dotplot synteny between chromosomes constructed with Hi-C data (x-axis) and scaffolds of the linkage groups (y-axis). **5**

Supplementary Figure 5. Karyotype of a female of *Megaleporinus macrocephalus* under C-banding. **6**

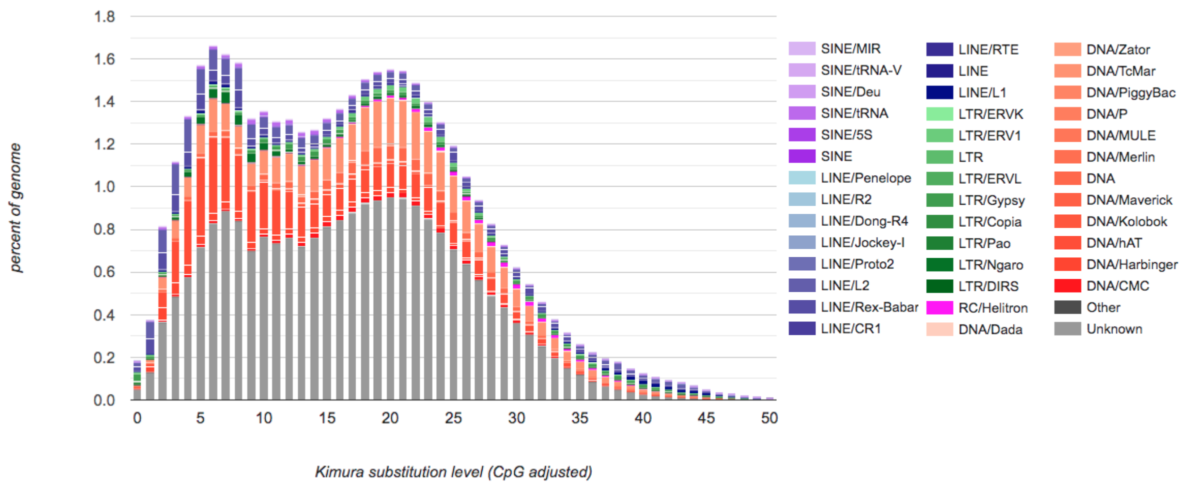

Supplementary Figure 1. Interspersed repeat landscape of *Megaleporinus macrocephalus* genome. The graph represents genome coverage (*y* axis) for each type of TEs (DNA transposons, SINE, LINE, and LTR retrotransposons), clustered according to Kimura distances (*K*-value; Kimura, 1980) to their corresponding consensus sequence (*x* axis, *K*-values from 0 to 50).

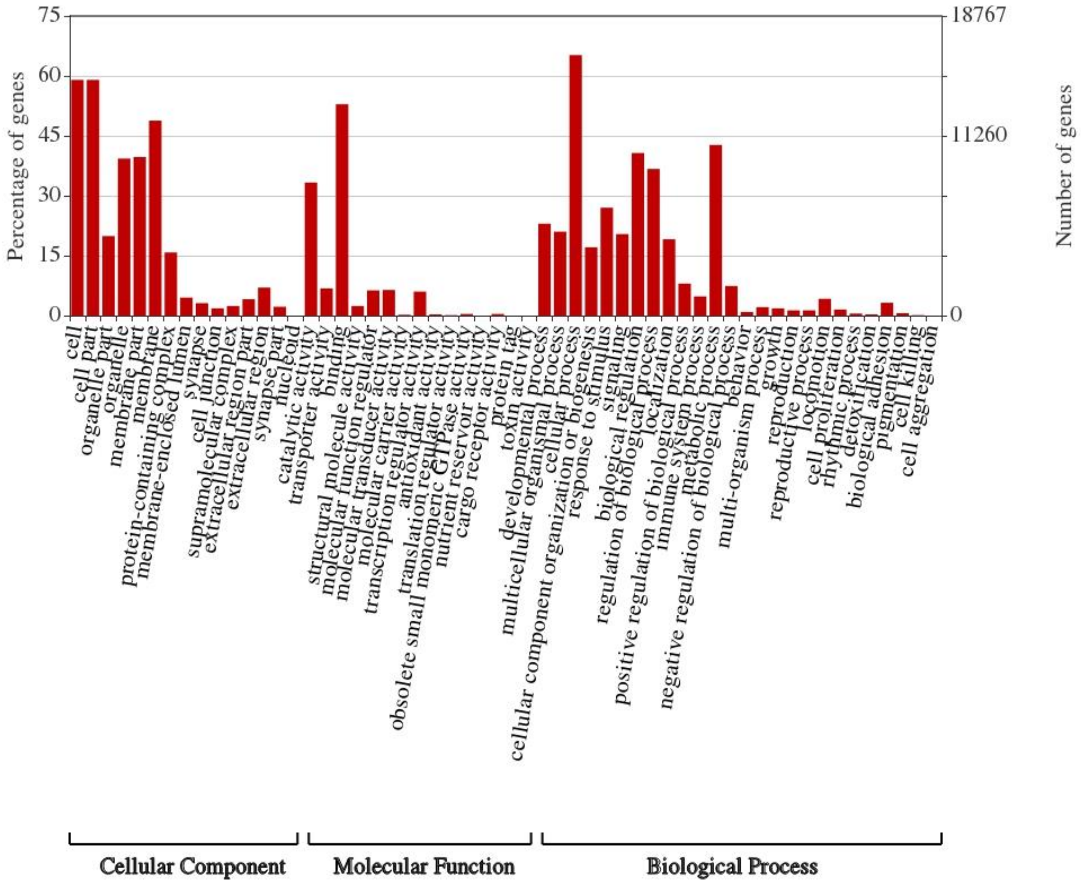

Supplementary Figure 2. Gene Ontology (G.O) Terms of Cellular Component, Molecular Function and Biological Process domains of *Megaleporinus macrocephalus* genome.

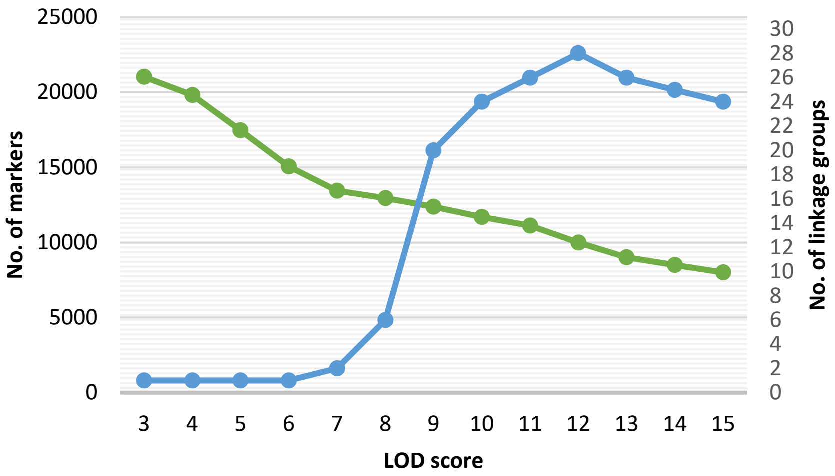

Supplementary Figure 3. The average number of markers in linkage groups (left y axis) and the number of linkage groups (right *y* axis) according to LOD score (*x* axis).

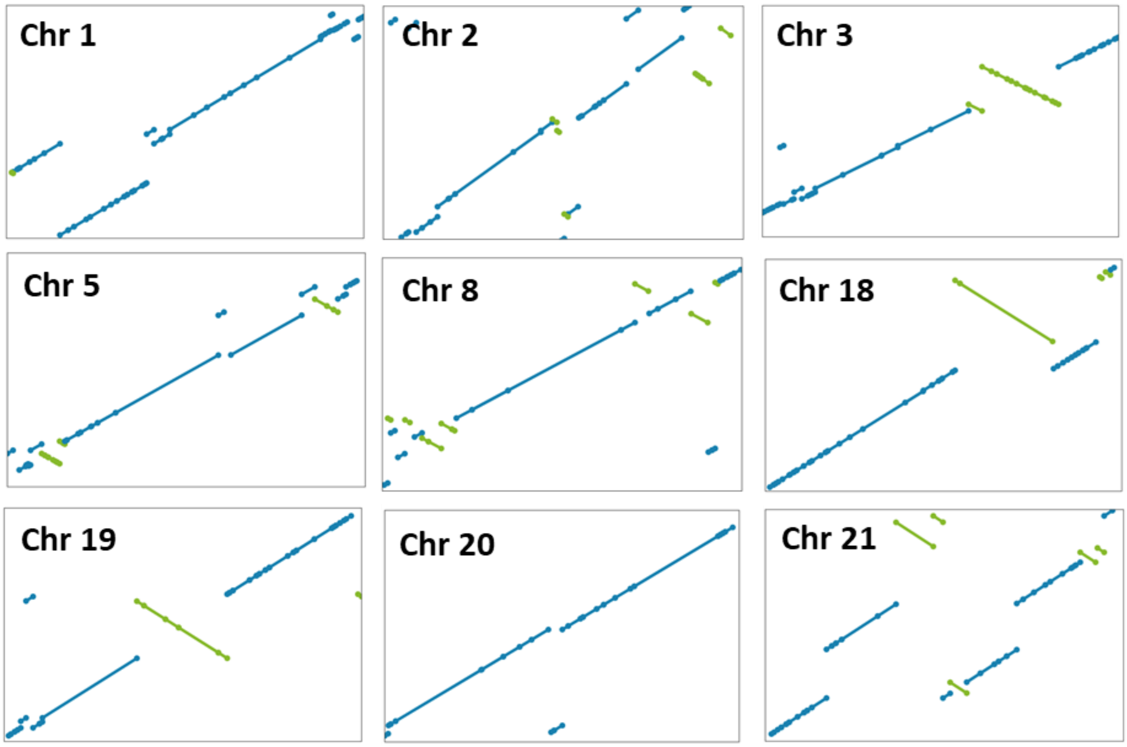

Supplementary Figure 4. Dotplot synteny between chromosomes constructed with Hi-C data (*x*-axis) and scaffolds of the linkage groups (*y*-axis). In blue, forward alignments; and in green, reverse alignments (inversions). The dots represent the end of scaffolds.

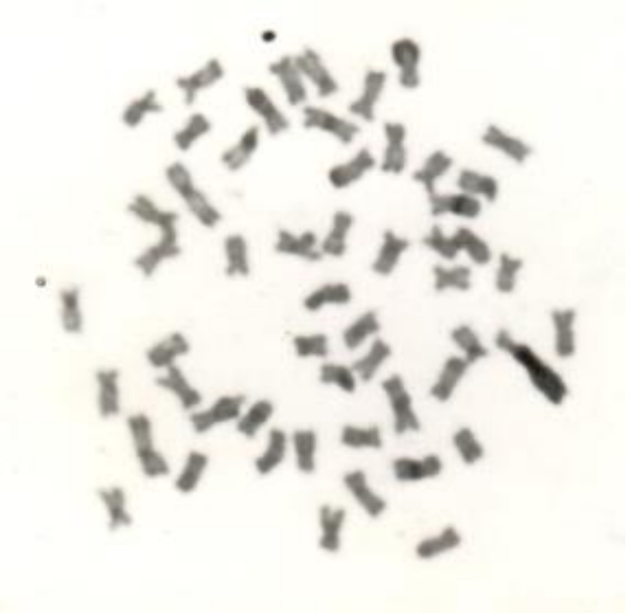

Supplementary Figure 5. Karyotype of a female of *Megaleporinus macrocephalus* under C-banding.
