# Supplementary material for: De novo assembly and characterization of a highly degenerated ZW sex chromosome in the fish Megaleporinus macrocephalus
Source: Gigascience. 2024 Nov 26;13:giae085. doi: 10.1093/gigascience/giae085 (PMC11590113; doi:10.1093/gigascience/giae085)
Supplement: giae085_supplement_Files [file giae085_supplement_files.zip › supplementary_material_tables.docx]

Summary

[Supplementary Table 1. Assembled and estimated chromosomes sizes (bp) calculated using karyotype data. 2](#_Toc155979887)

[Supplementary Table 2. Repeat annotation statistics for *Megaleporinus macrocephalus* genome. 3](#_Toc155979888)

[Supplementary Table 3. Summary of the annotated features of *Megaleporinus macrocephalus* genome 4](#_Toc155979889)

[Supplementary Table 4. Summary of ddRAD sequencing statistics. 5](#_Toc155979891)

[Supplementary Table 5. Summary of the genetic map of piauçu. Chr represents the chromosome which the linkage group had synteny with. Size is related to the length in bp, after scaffolding with Chromonomer. N is the number of markers. Length in cM, Density in cM/Locus. 6](#_Toc155979892)

[Supplementary Table 6. Summary of RNA-sequencing statistics. 8](#_Toc155979893)

[Supplementary Table 7. Top 10 genes up-regulated in females ZW (pink) and males ZZ (blue). 9](#_Toc155979894)

[Supplementary Table 8. Comparison between available genome assemblies of Neotropical fish species 10](#_Toc155979895)

[Supplementary Table 9. Statistics of a few Neotropical fish species linkage maps 11](#_Toc155979896)

[Supplementary Table 10. Summary of the individuals used for linkage map construction. 12](#_Toc155979897)

# Supplementary Table 1. Assembled and estimated chromosomes sizes (bp) calculated using karyotype data.

| **Autosome** | **Assembled size (bp)** | **Estimated size (bp)** |
| --- | --- | --- |
| 1 | 73,843,892 | 73,524,246 |
| 2 | 57,073,771 | 58,279,251 |
| 3 | 54,318,282 | 52,872,252 |
| 4 | 53,925,970 | 52,480,849 |
| 5 | 53,011,304 | 51,891,237 |
| 6 | 52,262,800 | 51,390,179 |
| 7 | 51,642,936 | 49,819,898 |
| 8 | 49,518,186 | 49,300,333 |
| 9 | 49,298,696 | 48,760,706 |
| 10 | 48,705,945 | 48,068,703 |
| 11 | 48,550,980 | 48,064,379 |
| 12 | 47,357,970 | 47,656,718 |
| 14 | 44,903,196 | 47,083,191 |
| 15 | 44,847,474 | 47,013,143 |
| 16 | 44,428,828 | 46,953,300 |
| 17 | 44,393,724 | 46,065,509 |
| 18 | 44,386,724 | 44,600,904 |
| 19 | 43,994,550 | 43,546,728 |
| 20 | 43,858,395 | 42,646,484 |
| 21 | 43,700,133 | 41,890,833 |
| 22 | 41,427,154 | 41,198,311 |
| 23 | 40,492,453 | 41,016,705 |
| 24 | 40,120,662 | 40,253,271 |
| 25 | 40,052,844 | 40,168,695 |
| 26 | 38,727,815 | 39,137,695 |
| 27 | 36,538,851 | 38,981,687 |
| **Pearson** | **0,99** |  |

# Supplementary Table 2. Repeat annotation statistics for *Megaleporinus macrocephalus* genome.

|  | **No. of elements** | **Length**  **(bp)** | **% of genome sequence** |
| --- | --- | --- | --- |
| **Retroelements** | 325,354 | 86,900,511 | 6.78 |
| SINEs: | 32,263 | 4,249,898 | 0.33 |
| Penelope | 1,992 | 200,769 | 0.02 |
| LINEs: | 135,677 | 43,882,452 | 3.42 |
| L2/CR1/Rex | 109,233 | 36,159,292 | 2.82 |
| R1/LOA/Jockey | 538 | 129,887 | 0.01 |
| R2/R4/NeSL | 222 | 69,099 | 0.01 |
| RTE/Bov-B | 10,276 | 2,603,408 | 0.20 |
| L1/CIN4 | 10,838 | 3,907,815 | 0.30 |
| LTR elements: | 157,414 | 38,768,161 | 3.02 |
| BEL/Pao | 2,206 | 845,394 | 0.07 |
| Ty1/Copia | 421 | 198,340 | 0.02 |
| Gypsy/DIRS1 | 24,957 | 7,950,002 | 0.62 |
| Retroviral | 10,995 | 2,687,534 | 0.21 |
| **DNA transposons** | 817,030 | 151,501,452 | 11.82 |
| hobo-Activator | 295,340 | 56,805,942 | 4.43 |
| Tc1-IS630-Pogo | 253,381 | 56,461,503 | 4.40 |
| PiggyBac | 3,214 | 595,853 | 0.05 |
| Tourist/Harbinger | 34,271 | 6,807,497 | 0.53 |
| Other  (Mirage, P-element, Transib) | 333 | 20,280 | 0.00 |
| Rolling-circles | 18,420 | 4248,326 | 0.33 |
| **Unclassified:** | 1379224 | 242,219,098 | 18.89 |
| **Total interspersed repeats:** |  | 480,621,061 | 37.49 |
| **Small RNA:** | 2375 | 321,707 | 0.03 |
| **Satellites:** | 88059 | 56,424,759 | 4.40 |
| **Simple repeats:** | 580511 | 52,069,796 | 4.06 |
| **Low complexity:** | 55387 | 5,150,938 | 0.40 |

Supplementary Table 3. Summary of the annotated features of *Megaleporinus macrocephalus* genome**.**

| **Feature** | ***Megaleporinus macrocephalus*** |
| --- | --- |
| **Genes and Pseudogenes** | 30,501 |
| **Protein-Coding** | 30,501 |
| **Exon** | 248,235 |
| **Intron** | 217,739 |
| **CDS** | 248,235 |
| **mRNA** | 30,501 |
| **Start codon** | 30,487 |
| **Stop codon** | 30, 488 |
| **Mean intron per gene** | 7.14 |
| **Mean exon per gene** | 8.14 |

| **Library** | **Total**  **sequences** | **Filtered sequences (%)** | | | | **Retained**  **reads**  **(%)** | **Average**  **retained reads/**  **ind.**  **(million)** |
| --- | --- | --- | --- | --- | --- | --- | --- |
|  |  | **Adapter**  **sequence** | **No**  **barcode** | **Low**  **quality** | **No**  **rad**  **cutside** |  |  |
| **1** | 198,679,472 | 1.19 | 3.67 | 2.37 | 1.33 | 91.44 | 3.95 |
| **2** | 191,095,010 | 1.18 | 4.92 | 2.43 | 1.66 | 89.82 | 3.73 |
| **3** | 183,389,588 | 1.15 | 4.36 | 2.59 | 1.86 | 90.04 | 3.59 |
| **4** | 188,028,608 | 1.17 | 4.38 | 2.53 | 1.81 | 90.12 | 3.68 |
| **5** | 165,196,674 | 1.09 | 4.53 | 2.42 | 1.70 | 90.27 | 3.24 |
| **6** | 207,410,674 | 1.15 | 6.49 | 2.32 | 1.86 | 88.18 | 3.98 |
| **7** | 173,700,306 | 1.15 | 6.88 | 2.76 | 2.07 | 87.14 | 3.29 |
| **Total** | 1,307,500,332 | 1.15 | 5.03 | 2.48 | 1.75 | 89.58 | 25.46 |

#

# Supplementary Table 4. Summary of ddRAD sequencing statistics.

# Supplementary Table 5. Summary of the genetic map of piauçu. Chr represents the chromosome which the linkage group had synteny with. Size is related to the length in bp, after scaffolding with Chromonomer. N is the number of markers. Length in cM, Density in cM/Locus.

| **LG** | **Chr** | **Size** | **n** | **Sex-averaged** | | **Male** | | **Female** | | **M:F** |
| --- | --- | --- | --- | --- | --- | --- | --- | --- | --- | --- |
|  |  |  |  | **Length** | **Density** | **Length** | **Density** | **Length** | **Density** |  |
| **1** | 6 | 23,599,497 | 710 | 126.42 | 0.18 | 135.77 | 0.19 | 131.68 | 0.19 | 1.03 |
| **2** | 4 | 45,953,104 | 688 | 130.95 | 0.19 | 127.07 | 0.18 | 107.22 | 0.16 | 1.19 |
| **3** | 1 | 49,978,048 | 625 | 133.56 | 0.21 | 139.93 | 0.22 | 130.11 | 0.21 | 1.08 |
| **4** | 15 | 40,070,618 | 543 | 120.91 | 0.22 | 139.69 | 0.26 | 133.3 | 0.25 | 1.05 |
| **5** | 10 | 43,303,417 | 476 | 122.43 | 0.26 | 106.81 | 0.22 | 100.3 | 0.21 | 1.06 |
| **6** | 18 | 34,104,434 | 484 | 112.29 | 0.23 | 135.78 | 0.28 | 115.72 | 0.24 | 1.17 |
| **7** | 2 | 50,146,178 | 489 | 129.75 | 0.27 | 156.05 | 0.32 | 121.16 | 0.25 | 1.29 |
| **8** | 9 | 44,463,537 | 503 | 107.79 | 0.21 | 116.85 | 0.23 | 115.32 | 0.23 | 1.01 |
| **9** | 25 | 36,288,099 | 429 | 135.49 | 0.32 | 137.46 | 0.32 | 135.06 | 0.31 | 1.02 |
| **10** | 23 | 36,576,158 | 501 | 122.64 | 0.24 | 152.38 | 0.3 | 117.92 | 0.24 | 1.29 |
| **11** | 16 | 32,187,476 | 444 | 111.72 | 0.25 | 108.1 | 0.24 | 80.47 | 0.18 | 1.34 |
| **12** | 8 | 43,378,694 | 442 | 127.77 | 0.29 | 136.22 | 0.31 | 99.65 | 0.23 | 1.37 |
| **13** | 11 | 36,977,433 | 473 | 121.11 | 0.26 | 128.1 | 0.27 | 124.15 | 0.26 | 1.03 |
| **14** | 12 | 44,454,369 | 406 | 141.52 | 0.35 | 142.35 | 0.35 | 135.23 | 0.33 | 1.05 |
| **15** | 27 | 35,428,719 | 381 | 119.88 | 0.31 | 120.71 | 0.32 | 119.61 | 0.31 | 1.01 |
| **16** | 17 | 38,667,919 | 372 | 139.89 | 0.38 | 139.95 | 0.38 | 141.94 | 0.38 | 0.99 |
| **17** | 19 | 25,433,675 | 385 | 121.22 | 0.31 | 124.13 | 0.32 | 133.74 | 0.35 | 0.93 |
| **18** | 24 | 19,470,523 | 339 | 119.57 | 0.35 | 121.55 | 0.36 | 121.27 | 0.36 | 1 |
| **19** | 22 | 27,071,565 | 351 | 119.33 | 0.34 | 137.5 | 0.39 | 121.55 | 0.35 | 1.13 |
| **20** | 3 | 38,970,815 | 296 | 82.15 | 0.28 | 94.36 | 0.32 | 95.58 | 0.32 | 0.99 |
| **21** | 7 | 18,150,482 | 253 | 81.57 | 0.32 | 96.9 | 0.38 | 92.98 | 0.37 | 1.04 |
| **22** | 20 | 39,059,067 | 236 | 143.08 | 0.61 | 142.45 | 0.6 | 130.32 | 0.55 | 1.09 |
| **23** | 21 | 26,787,905 | 250 | 107.64 | 0.43 | 111.77 | 0.45 | 104.11 | 0.42 | 1.07 |
| **24** | 13 | 4,059,682 | 225 | 43.25 | 0.19 | 47.37 | 0.21 | 87.81 | 0.39 | 0.54 |
| **25** | 5 | 23,599,497 | 237 | 132.10 | 0.56 | 150.95 | 0.64 | 139.78 | 0.59 | 1.08 |
| **26** | 14 | 45,953,104 | 239 | 136.79 | 0.57 | 135.83 | 0.57 | 147.43 | 0.62 | 0.92 |
| **27** | 13 | 22,889,721 | 251 | 120.66 | 0.48 | 117.89 | 0.47 | 113.62 | 0.45 | 1.04 |
| **28** | 26 | 29,510,817 | 203 | 108.88 | 0.54 | 114.34 | 0.56 | 104.98 | 0.52 | 1.09 |
| **Total** |  | 977,082,963 | 11,231 | 3320.36 | 0.29 | 3,518.24 | 0.31 | 3,301.97 | 0.29 | 1.07 |

# Supplementary Table 6. Summary of RNA-sequencing statistics.

| **Pool** | **Replicate** | **Total reads** | **Retained reads (%)** | **Total data (Gb)** |
| --- | --- | --- | --- | --- |
| **Male** | 1 | 46,331,866 | 97.72 | 4.53 |
|  | 2 | 50,141,272 | 97.93 | 4.91 |
|  | 3 | 41,915,706 | 100.00 | 4.44 |
| **Female** | 1 | 50,886,670 | 97.58 | 4.97 |
|  | 2 | 48,466,868 | 97.21 | 4.71 |
|  | 3 | 48,337,546 | 97.33 | 4.70 |
|  | **Total** | 286,079,928 | 97.91 | 28.26 |

# Supplementary Table 7. Top 10 genes up-regulated in females ZW (pink) and males ZZ (blue).

| **transcript ID** | **LFC** | ***p*adj** | **chr** | **gene ID** | **description** |
| --- | --- | --- | --- | --- | --- |
| Mmac_g35407.t1 | -12.73 | 2.57E-13 | chr6 | *-* | Zona pellucida sperm-binding protein 3-like |
| Mmac_g39735.t2 | -12.28 | 2.79E-82 | chr9 | *-* | Zona pellucida sperm-binding protein 3-like |
| Mmac_g34977.t1 | -11.75 | 1.19E-08 | chr6 | *Zp4* | Zona pellucida |
| Mmac_g34976.t2 | -11.57 | 3.05E-14 | chr6 | *Zp4* | Zona pellucida |
| Mmac_g21232.t2 | -11.54 | 1.54E-05 | chr21 | *-* | Zona pellucida sperm-binding protein 3-like |
| Mmac_g36656.t1 | -11.24 | 1.26E-61 | chr7 | *-* | Zona pellucida sperm-binding protein 3-like |
| Mmac_g18381.t1 | -11.13 | 2.41E-33 | chr2 | *-* | Zona pellucida sperm-binding protein 3-like |
| Mmac_g14296.t1 | -10.99 | 2.45E-58 | chr18 | *-* | Zona pellucida sperm-binding protein 3-like |
| Mmac_g36128.t1 | -10.92 | 1.07E-16 | chr7 | *Aqp1* | Belongs to the MIP aquaporin (TC 1.A.8) family |
| Mmac_g7248.t1 | -10.88 | 3.09E-12 | chr13 | *Smarcd1* | SWI SNF related, matrix associated, actin dependent regulator of chromatin, subfamily d, member 1 |
| Mmac_g2999.t1 | 9.31 | 1.75E-54 | chr10 | *Ces5a* | Belongs to the type-B carboxylesterase lipase family |
| Mmac_g33548.t1 | 6.67 | 3.80E-32 | chr5 | *Enpep* | Glutamyl aminopeptidase |
| Mmac_g22869.t1 | 8.50 | 6.87E-26 | chr22 | *-* | Insulin / insulin-like growth factor / relaxin family. |
| Mmac_g38128.t1 | 9.00 | 4.30E-24 | chr8 | *Ppp1r1c* | Protein phosphatase 1 regulatory |
| Mmac_g5032.t1 | 6.79 | 1.53E-23 | chr11 | *Coch* | Coagulation factor C homolog. cochlin (*Limulus polyphemus*) |
| Mmac_g12538.t1 | 6.78 | 1.53E-23 | chr16 | *-* | Gonadal somatic cell derived factor |
| Mmac_g4256.t1 | 7.03 | 1.55E-22 | chr11 | *Fabp7* | Belongs to the calycin superfamily. Fatty-acid binding protein (FABP) family |
| Mmac_g19985.t1 | 6.30 | 7.59E-21 | chr20 | *Amh* | Anti-mullerian hormone |
| Mmac_g39628.t1 | 6.39 | 3.34E-20 | chr9 | *-* | Apelin receptor |
| Mmac_g25145.t1 | 6.20 | 1.14E-18 | chr24 | *Tbx1* | T-box transcription factor |

# Supplementary Table 8. Comparison between available genome assemblies of Neotropical fish species

| **Species** | **Genome size*(bp)** | **Scaffold N50 (bp)** | **Contig N50 (bp)** | **Haploid chromosome number (n)** |
| --- | --- | --- | --- | --- |
| *Megaleporinus macrocephalus*  (GCA_021613375.1) | 1,280,781,66 | 45,034,219 | 5,013,076 | 27 |
| *Colossoma macropomum*  (GCA_904425465.1) | 1,221,809,066 | 40,163,545 | 5,645,235 | - |
| *Pygocentrus nattereri*  (GCA_015220715.1) | 1,222,050,449 | 42,283,192 | 12,898,870 | 30 |
| *Astyanax mexicanus*  (GCA_000372685.2) | 1,291,596,431 | 35,377,769 | 1,767,240 | 25 |

*Ungapped length

Supplementary Table 9. Statistics of a few Neotropical fish species linkage maps**.**

| **Species** | **Technique** | **No. of SNPs** | **Length**  **(cM)** | **Average marker interval (cM)** | **Reference** |
| --- | --- | --- | --- | --- | --- |
| *Megaleporinus macrocephalus* | ddRADseq | 11,231 | 3,320.36 | 0.29 | This study |
| *Colossoma macropomum* | GBS* | 7,734 | 2,811 | 0.39 | Nunes *et al*., 2017 |
|  | RADseq | 14,805 | 2,752 | 0.51 | Varela et al., 2021 |
| *Piaractus mesopotamicus* | RADseq | 17,453 | 2,755.60 | 0.47 | Mastrochirico-Filho *et al*., 2020 |

*Genotype-by-sequencing

# Supplementary Table 10. Summary of the individuals used for linkage map construction.

| Family | Males | Females | Total |
| --- | --- | --- | --- |
| 1 | 49 | 44 | 93 |
| 2 | 4 | 20 | 24 |
| 3 | 49 | 44 | 93 |
| 4 | 41 | 48 | 89 |
| Total | 143 | 156 | 299 |
