# Supplementary material for: De novo assembly and characterization of a highly degenerated ZW sex chromosome in the fish Megaleporinus macrocephalus
Source: Gigascience. 2024 Nov 26;13:giae085. doi: 10.1093/gigascience/giae085 (PMC11590113; doi:10.1093/gigascience/giae085)
Supplement: giae085_GIGA-D-24-00015_Revision_2 [file giae085_giga-d-24-00015_revision_2.pdf]

GigaScience

De novo assembly and characterization of a highly degenerated ZW sex chromosome in the fish Megaleporinus macrocephalus

--Manuscript Draft--

Manuscript Number:	GIGA-D-24-00015R2
Full Title:	De novo assembly and characterization of a highly degenerated ZW sex chromosome in the fish Megaleporinus macrocephalus
Article Type:	Data Note
Funding Information:	Coordenação de Aperfeiçoamento de Pessoal de Nível Superior (88887.467255/2019-00) — Ms. Carolina Heloisa de Souza Borges<br>Governo Brasil — Not applicable<br>Conselho Nacional de Desenvolvimento Científico e Tecnológico (404386/2021) — Mrs. Fausto Foresti
Abstract:	Background<br>Megaleporinus macrocephalus (piauçu) is a Neotropical fish within Characoidei that presents a well-established heteromorphic ZZ/ZW sex-determination system and thus, constitutes a good model for studying W and Z chromosomes in fishes. We used PacBio reads and Hi-C to assemble a chromosome-level reference genome for M. macrocephalus. We generated family segregation information to construct a genetic map, pool-seq of males and females to characterize its sex system, and RNA-seq to highlight candidate genes of M. macrocephalus sex determination.<br><br>Results<br>The reference genome of M. macrocephalus is 1,282,030,339 bp in length and has a contig and scaffold N50 of 5.0 Mb and 45.03 Mb, respectively. In the sex chromosome, based on patterns of recombination suppression, coverage, Fst, and sex-specific SNPs, we distinguished a putative W-specific region that is highly differentiated, a region where Z and W still share some similarities and is undergoing degeneration, and the PAR. The sex chromosome gene repertoire includes genes from the TGF-β family (amhr2, bmp7) and the Wnt/β-catenin pathway (wnt4, wnt7a), some of which are differentially expressed.<br><br>Conclusions<br>The chromosome-level genome of piauçu exhibits high quality, establishing a valuable resource for advancing research within the group. Our discoveries offer insights into the evolutionary dynamics of Z and W sex chromosomes in fish, emphasizing ongoing degenerative processes and indicating complex interactions between Z and W sequences in specific genomic regions. Notably, amhr2 and bmp7 are potential candidate genes for sex determination in M. macrocephalus.<br><br>Keywords: chromosome-level genome; sex chromosome assembly; amhr2; sex determination
Corresponding Author:	Diogo Teruo Hashimoto, Ph.D<br>UNESP: Universidade Estadual Paulista Julio de Mesquita Filho<br>Jaboticabal, São Paulo BRAZIL
Corresponding Author Secondary Information:	
Corresponding Author's Institution:	UNESP: Universidade Estadual Paulista Julio de Mesquita Filho
Corresponding Author's Secondary Institution:	
First Author:	Carolina Heloisa de Souza Borges, Ph.D
First Author Secondary Information:	

Powered by Editorial Manager® and ProduXion Manager® from Aries Systems Corporation

**Order of Authors:**	Carolina Heloisa de Souza Borges, Ph.D
	Ricardo Utsunomia
	Alessandro de Mello Varani, Ph.D
	Marcela Uliano-Silva
	Lieschen Valeria Guerra Lira, Ph.D
	Arno Juliano Butzge
	John Fredy Gomez Agudelo
	Shisley Cristina da Silva Manso
	Milena Vieira de Freitas
	Raquel Belini Ariede
	Vito Antonio Mastrochirico-Filho
	Carolina Penaloza
	Agustin Barria
	Fabio Porto-Foresti
	Fausto Foresti
	Ricardo Hattori
	Yann Guiguen
	Ross D. Houston
	Diogo Teruo Hashimoto, Ph.D
**Order of Authors Secondary Information:**	
**Response to Reviewers:**	Reviewer reports:<br>Reviewer #2: This study assembled the genome of Megaleporinus macrocephalus, identified the sex chromosomes and the boundaries of pseudoautosomal region and Z/W-specific region, and statistically analyzed the distribution of genes that are differentially expressed in male and female gonads in sex chromosome. It provides a theoretical foundation for the study of heteromorphic sex chromosomes in fish. I suggest making few revisions to the article as a whole.<br><br>1."These species were formerly classified under the genus Leporinus but were reclassified due to the presence of a unique ZZ/ZW sex chromosome system that emerged at least 12 million years ago" Give examples of species with a ZZ/ZW sex chromosome system and explain whether they have homomorphic or heteromorphic sex chromosomes.<br>Please find the modifications in lines 9-15.<br><br>2."it remains the only well-characterized W chromosome in fish" It is not only. The homomorphic Z/W chromosomes of Verasper variegatus has published.<br>Thank you for the comment. The modified text is in lines 55-58.<br><br>3.Does M. macrocephalus exhibit sexual dimorphism, and what economic value does this study foresee for it?<br>Please see the answer in lines 17-19 and 65-67 of the Background section.<br><br>4."The unique molecular yield of PacBio reads was 56 Gb, and the subread N50 length was 32 kb." The first half of the sentence refers to the number of reads, right? If so, the unit "Gb" is not appropriate to use.<br>Thank you for your comment; you are correct. We have removed the sentence to improve the clarity of the paragraph.<br><br>5.The content from lines 77 to 80 can be moved to the Methods section.<br>Thank you for your suggestion. We have moved the content to the Methods section, and the modifications can be found in lines 628-630.

Powered by Editorial Manager® and ProduXion Manager® from Aries Systems Corporation

	6.The content from lines 95 to 102 is too verbose. It could serve as a caption for Figure 1B.<br>Thank you for your suggestion. The content has been moved to the caption of Figure 1B, and the changes can be found in lines 96-105.<br><br>7."as both LG24 and LG27 correspond to different regions of the same sex chromosome." However, as can be seen from Figure 3B, part of LG24 also shows collinearity with some regions of chromosomes 25 and 27. This does not adequately prove that LG24 is part of the sex chromosome; it also includes parts of the autosomes.<br>Thank you for your comment! We have addressed this issue and added the relevant information in lines 238-240.<br><br>8."which is undergoing degeneration." What results could corroborate this conclusion?<br>Thank you for your observation. We have included the supporting information in lines 324-326.<br><br>9.Based on information such as linkage groups and defined regions, attempt to assemble the Z and W chromosomes.<br>We greatly appreciated this suggestion. However, after careful analysis, we decided not to proceed with reassembling the Z and W chromosomes, as it would result in spurious assemblies. This discussion has been included in the Discussion section (lines 523-557).
**Additional Information:**	
**Question**	**Response**
Are you submitting this manuscript to a special series or article collection?	No
**Experimental design and statistics**<br><br>Full details of the experimental design and statistical methods used should be given in the Methods section, as detailed in our Minimum Standards Reporting Checklist. Information essential to interpreting the data presented should be made available in the figure legends.<br><br>Have you included all the information requested in your manuscript?	Yes
**Resources**<br><br>A description of all resources used, including antibodies, cell lines, animals and software tools, with enough information to allow them to be uniquely identified, should be included in the Methods section. Authors are strongly encouraged to cite Research Resource Identifiers (RRIDs) for antibodies, model organisms and tools, where possible.	Yes

Have you included the information requested as detailed in our Minimum Standards Reporting Checklist?	
**Availability of data and materials**<br><br>All datasets and code on which the conclusions of the paper rely must be either included in your submission or deposited in publicly available repositories (where available and ethically appropriate), referencing such data using a unique identifier in the references and in the “Availability of Data and Materials” section of your manuscript.<br><br>Have you have met the above requirement as detailed in our Minimum Standards Reporting Checklist?	Yes

Powered by Editorial Manager® and ProduXion Manager® from Aries Systems Corporation

Manuscript

Click here to access/download;Manuscript;artigo_Megaleporinus_submissio

De novo assembly and characterization of a highly degenerated ZW sex chromosome in the fish *Megaleporinus macrocephalus*

Carolina Heloisa de Souza Borges[1], Ricardo Utsunomia[2], Alessandro Varani[3], Marcela Uliano-Silva[4], Lieschen Valeria G. Lira[1], Arno J. Butzge[1], John F. Gomez Agudelo[1], Shisley Manso[1], Milena V. Freitas[1], Raquel B. Ariede[1], Vito A. Mastrochirico-Filho[1], Carolina Penaloza[5], Agustín Barria[5], Fábio Porto-Foresti[2], Fausto Foresti[6], Ricardo Hattori[7], Yann Guiguen[8], Ross D. Houston[5] and Diogo Teruo Hashimoto[1]

[1]*São Paulo State University (Unesp), Aquaculture Center of Unesp, Jaboticabal, SP, Brazil*

[2]*São Paulo State University (Unesp), School of Sciences, Bauru, SP, Brazil*

[3]*São Paulo State University (Unesp), School of Agricultural and Veterinary Sciences, Jaboticabal, SP, Brazil*

[4]*Welcome Sanger Institute, Cambridge, United Kingdom*

[5]*University of Edinburgh, The Roslin Institute, Easter Bush, Midlothian, United Kingdom*

[6]*São Paulo State University (Unesp), Institute of Biosciences, Botucatu, SP, Brazil*

[7] *Sao Paulo Agency of Agribusiness and Technology (APTA), São Paulo, SP, Brazil*

[8]*INRAE, LPGP, Rennes, France*

Corresponding author: diogo.hashimoto@unesp.br

Carolina Heloisa de Souza Borges [0000-0002-6950-3082]; Ricardo Utsunomia [0000-0003-4565-6065]; Alessandro de Mello Varani [0000-0002-8876-3269]; Marcela Uliano-Silva [0000-0001-6723-4715]; Lieschen Valeria Guerra Lira [0000-0003-0971-001X]; Arno Juliano Butzge [0000-0002-4580-7145]; John Fredy Gomez Agudelo; Shisley Cristina da Silva Manso; Milena Vieira de Freitas [0000-0002-6434-2590]; Vito Antonio Mastrochirico-Filho [0000-

0002-3550-2671]; Carolina Penaloza [0000-0001-7052-3614]; Agustin Barria [0000-0002-2813-4559]; Fabio Porto-Foresti [0000-0001-8845-3845]; Fausto Foresti [0000-0002-0862-0445]; Ricardo Hattori [0000-0003-2605-6718]; Yann Guiguen [0000-0001-5464-6219]; Ross D Houston [0000-0003-1805-0762]; Diogo Teruo Hashimoto [0000-0002-8808-2498];

Abstract

Background

Megaleporinus macrocephalus (piauçu) is a Neotropical fish within Characoidei that presents a well-established heteromorphic ZZ/ZW sex-determination system and thus, constitutes a good model for studying W and Z chromosomes in fishes. We used PacBio reads and Hi-C to assemble a chromosome-level reference genome for *M. macrocephalus*. We generated family segregation information to construct a genetic map, pool-seq of males and females to characterize its sex system, and RNA-seq to highlight candidate genes of *M. macrocephalus* sex determination.

Results

The reference genome of *M. macrocephalus* is 1,282,030,339 bp in length and has a contig and scaffold N50 of 5.0 Mb and 45.03 Mb, respectively. In the sex chromosome, based on patterns of recombination suppression, coverage, F_{st}, and sex-specific SNPs, we distinguished a putative W-specific region that is highly differentiated, a region where Z and W still share some similarities and is undergoing degeneration, and the PAR. The sex chromosome gene repertoire includes genes from the TGF-β family (*amhr2*, *bmp7*) and the Wnt/β-catenin pathway (*wnt4*, *wnt7a*), some of which are differentially expressed.

Conclusions

The chromosome-level genome of piauçu exhibits high quality, establishing a valuable resource for advancing research within the group. Our discoveries offer insights into the evolutionary dynamics of Z and W sex chromosomes in fish, emphasizing ongoing degenerative processes and indicating complex interactions between Z and W sequences in specific genomic regions. Notably, *amhr2* and *bmp7* are potential candidate genes for sex determination in *M. macrocephalus*.

Keywords: chromosome-level genome; sex chromosome assembly; *amhr2*; sex determination

1. Background

The family Anostomidae, native to the Neotropical region, comprises 147 recognized species across 16 genera [1], distributed from northern Colombia to the La Plata River in Argentina [2]. Among these, one of the most economically important genus is *Megaleporinus*, composed of relatively large species, with adults typically exceeding 35 cm in standard length [3]. *Megaleporinus* species were previously classified under *Leporinus*, but were reclassified due to the presence of a unique ZZ/ZW sex chromosome system that originated at least 12 million years ago [4].

Historically, cytogenetic studies on *Leporinus* species have shown that most species, including *Leporinus friderici*, *L. fasciatus* and *L. octomaculatus*, possess cytologically indistinguishable, or 'homomorphic,' [5] sex chromosomes [6] [7]. In contrast, some species—such as *L. conirostris, L. macrocephalus, L. obtusidens, L. reinhardti,* and *L. trifasciatus*—exhibit differentiated, or 'heteromorphic,' [5] sex chromosomes [8] [7] [9]. This feature was proposed as a synapomorphy for a potential monophyletic group [8], later named *Megaleporinus* [4].

Known in Brazil as ‘piauçu’, *Megaleporinus macrocephalus* (NCBI:txid327003; marinespecies.org:taxname:1524119) is an omnivorous anostomid that consumes small fruits, seeds, small fish, and crabs [10]. This species is found in the Paraguay River [11] and exhibits sexual size dimorphism, with females being larger than males [3]. Moreover, it is the only aquaculture species in Brazil with a well-established ZZ/ZW heteromorphic sex chromosome system [12]. The W chromosome is the largest in its karyotype, with its long arms being entirely heterochromatic. In contrast, the Z chromosome is a medium-sized metacentric chromosome with small portions of heterochromatin confined to the ends of its long arms [7].

Currently, there are limited genomic resources available for piauçu in public databases. These resources consist mainly of fragments of mitochondrial and nuclear genes and

microsatellite sequences used for genetic monitoring in aquaculture [13], species identification [14] [15] [16], phylogenetic analyses [15] [17] [18], and population studies [19]. To our knowledge, there is no reference genome available for this species.

Although the sex chromosome system of *M. macrocephalus* was identified cytologically over 40 years ago [7], the current knowledge of the Z and W chromosomes is limited to repetitive sequences, particularly from a chromosome-scale perspective, *i.e.*, there is no in-depth information about the genomic structure (non-recombining and pseudoautosomal regions), gene contents, transcription status, and patterns of recombination. The species exhibits a significant expansion of satellite DNA (satDNA) in its genome compared to other Characoidei fish [20] [21] [22]. This expansion results from the duplication of existing satellites followed by substitution/deletion/insertion events, as well as other unknown mechanisms [12]. Through low-coverage sequencing of male and female individuals, [12] constructed a satellitome for the species and mapped sex-biased satellites using fluorescent in situ hybridization (FISH). Approximately 18% of the satDNAs had differentially accumulated within the heteromorphic sex chromosomes of *M. macrocephalus*, suggesting a high degree of differentiation between the Z and W chromosomes, likely due to the loss of recombination between these chromosomes [23]. As expected in a monophyletic ZZ/ZW system, they identified some satellites conserved in the W chromosomes of both *M. macrocephalus* and *Megaleporinus obtusidens*, indicating that these satellites were present in the common ancestor of these species before their evolutionary split. Moreover, there were satellites with differential accumulation or exclusive to the piauçu W chromosome, which highlights the occurrence of an independent and continuous differentiation of the W chromosomes in this genus.

While there have been disruptive advances in sequencing technologies for assembling high-quality genomes of non-model species, such as long-read sequencing and scaffolding techniques like Hi-C sequencing [24] [25] [26], sex chromosomes have been notoriously

difficult to assemble due to their high divergence in the heterogametic sex and high repeat content [27]. Recently, several new Y chromosome assemblies have been reported in fish, such as in the zig-zag eel [28], threespine stickleback [29], Atlantic herring [30], and the neo-Y chromosome of the spotted knifejaw [31]. However, there have been few reports of fish W chromosome assemblies. A decade ago, the first W chromosome assembly in fish was completed for the tongue sole, *Cynoglossus semilaevis* [32]. Since then, only a few well-characterized W chromosomes in fish have been made available, such as in *Verasper variegatus* [33].

The study of W chromosome evolution in vertebrates continues to be constrained by the scarcity of W assemblies; therefore, it is of paramount importance to sequence additional W models. *Megaleporinus macrocephalus* is an ideal model for studying the structure and evolution of Z and W chromosomes in fish as it belongs to a rare group with conserved ZW chromosomes. Understanding the detailed genomic architecture of these chromosomes can provide insights into mechanisms of sex chromosome differentiation and degeneration in fish. Furthermore, as an important species in Brazilian aquaculture, discovering sex-related genomic resources for *M. macrocephalus* can aid conservation efforts and breeding programs, supporting genomic selection and female-biased sex control in aquaculture. In this study, we aimed to: 1) assemble a chromosome-level genome of *Megaleporinus macrocephalus*, including the sex chromosome; 2) assess patterns of recombination in the sex chromosome by linkage mapping; 3) characterize the genomic regions of the sex chromosome by resequencing of male and female individuals; and 4) identify candidate genes for sex determination by RNA-seq experiments.

2. Results

2.1 Chromosome-level genome assembly

2.1.1 Genome Assembly

We generated 88.8 Gb of Pacific Biosciences (PacBio) continuous long reads (CLR), 85 Gb of MGISEQ short reads, and 105 Gb of Hi-C data. The genome coverage based on final assembly size was 69.4x, 66.4x, and 82x, respectively. After removing poor-quality sequences from the short reads, we retained 82 Gb of clean data. This dataset was used to generate *k*-mer spectrum plots to estimate the overall characteristics of the genome. All *k*-mer plots were similar and indicated a low heterozygosity rate (**Figure 1A**). The estimated genome size (based on 21-mer) was 1.02 Gb with a heterozygosity of 0.50% and 16.9% of repeat content.

PacBio long reads were assembled using Falcon/Falcon-Unzip Unzip [34], resulting in 2,770 primary contigs, including 33 contigs > 5 Mb, with an N50 of 1.53 Mb. After gap filling, the contigs were clustered into 1,227 scaffolds, achieving an N50 of 5.0 Mb. These scaffolds were then ordered and oriented into 27 chromosomes, consistent with the species' haploid chromosome number [35], along with 73 unplaced scaffolds (< 250 kb). The 27 chromosomes accounted for 99.56% of the complete genome assembly.

The final *M. macrocephalus* reference genome contains 27 chromosomes and 73 unplaced scaffolds. It has a contig and scaffold N50 of 5.0 Mb and 45.03 Mb, respectively, with an assembled genome size of 1.28 Gb (**Table 1**).

Figure 1. (A) *K*-mer profile of MGISEQ short reads. (B) A *k*-mer analysis of the *Megaleporinus macrocephalus* genome bases against its sequenced MGISEQ reads. The results show that (i) the distribution of the *k*-mers in the assembly is consistent with the short read profile, (ii) two peaks are demonstrating that 1-copy (heterozygous) and 2-copy (homozygous) *k*-mers were found once in the assembly, as expected for a pseudo-haplotype genome [36], (iii) most of the assembly *k*-mers (in red) are unique, indicating that the assembly

has a low content of artificial duplications (*i.e.*, *k*-mers found twice, in blue) (iv) there are missing *k*-mers in the assembly (black peak), which is compatible with haploid genomes, (v) the 1-copy *k*-mer peak (red) is greater than its missing sequences (black), this suggests that Falcon-Unzip erroneously included sequences from both haplotypes into the primary pseudo-haplotype [36].

Table 1. Statistics for genome assembly of *Megaleporinus macrocephalus*.

characteristic	value
no. scaffolds	101
no. contigs	1,353
main genome scaffold sequence total (bp)	1,282,030,339
main genome contig sequence total (bp)	1,280,781,659
scaffold N50 (bp)	45,034,219
contig N50 (bp)	5,013,076
max. scaffold length (bp)	73,843,892
max. contig length (bp)	25,940,738
% main genome in scaffolds > 50 kb	99.9%
BUSCO complete	96.2%
BUSCO complete and single copy	95.1%
BUSCO complete and duplicated	1.1%
BUSCO fragmented	0.6%
BUSCO missing	3.2%
consensus quality value (QV)	37.53
merqury completeness	93.05%

We used the highly accurate short reads to plot Merqury [36] evaluation against the genome *k*-mers (**Figure 1B)**. The accuracy of the base calls (QV), which is calculated using the *k*-mers found only in the assembly (bar at the beginning of **Figure 1B**), was 37. 53 (**Table 1**) and represents a base accuracy > 99.9% (*e.g.*, QV = 30 means 99.9% accuracy). The

completeness score shows that 93.05% of *k*-mers in the MGISEQ reads are present in the assembly, which is a good recovery of *k*-mers for a species with 0.5% heterozygosity.

Pearson's correlation between the autosomes assembled size with its actual karyotypic size (**Supplementary Table 1**) was 99%, demonstrating the high quality of the assembled *M. macrocephalus* genome.

2.1.2 Sex chromosome

Chromosome 13 was recognized as the sex chromosome based on the following evidence:

- In the Hi-C contact map, we observed lower coverage in the upper segment of this chromosome compared to its terminal segment and other chromosomes. This upper segment is assumed to correspond to the W-specific region (hemizygous), while the terminal segment corresponds to the pseudo-autosomal region of the Z and W chromosomes (**Figure 2**).
- In the linkage map, the linkage group 24 (LG24) exhibited suppression of recombination, with varying intensities in the female and male maps (**Figure 4**). LG24 presented collinearity with chromosome 13 (**Figure 3B**).
- The comprehensive examination of SNP distribution through resequencing analysis revealed a robust sex-linked signal in females and elevated fixation index (F_{ST}) values within chromosome 13 (**see Figure 5**).

As expected, due to the PacBio CLR approach, which results in a high error rate (~15%) [37], it was not possible to determine the identity of the contigs (whether they are Z or W reads) belonging to the non-recombining region of the sex chromosomes. Consequently, accurate phased assembly of the sex chromosomes could not be achieved in this study. Thus, we cannot conclude whether chromosome 13 is the W chromosome or the Z chromosome. Therefore,

chromosome 13 is represented as a consensus of the Z and W chromosomes and will henceforth be referred to as the sex chromosome.

Figure 2. Hi-C contact map of *Megaleporinus macrocephalus* with the sex chromosome indicated by an arrow.

2.1.3 Repeat Annotation

Using the *de novo* prediction model, 2,544 new families of repeats were identified in the genome. The repeat content in *M. macrocephalus* accounted for 46.71% of the genome (598 Mb). Among the repeats, transposable elements were the most common, representing 37.49% of the genome. DNA transposons were the most abundant TEs (11.82%), followed by long terminal repeats (LTR) at 3.02%, long interspersed nuclear elements (LINE) at 3.42%, and short interspersed nuclear elements (SINEs) at 0.33% (**Supplementary Table 2**). A significant portion (18.89%) of the interspersed repeats remained unclassified. Despite using the species' satellitome [12] to identify the satellite DNAs, these repeats accounted for only 4.40% of the genome (**Supplementary Table 2**).

Supplementary Figure 1 shows older TE copies located on the right side of the graph, while more recent ones, which do not diverge much from the consensus TE sequence, are on the left side. Most of the interspersed repeat content found in the *M. macrocephalus* genome is recent (*K*-values < 25). Also, two bursts of transposition dominated by DNA transposon are observable.

The repeat content found in the sex chromosome was slightly higher than in the autosomes (4.24%). The total interspersed repeats and satellites classes showed the most significant differences, 2.37% and 2.24%, respectively (**Table 2**).

Table 2. Comparison between the repeat content in the sex chromosome and the autosomes of the *Megaleporinus macrocephalus* genome.

	**% sex chromosome**	**% autosomes**
repeat content	50.95	46.71
retroelements	7.86	6.78
DNA transposons	12.07	11.82
unclassified	19.92	18.89
total interspersed repeats	39.86	37.49
small RNA	0.02	0.03
satellites	6.64	4.40
simple repeats	3.72	4.06
low complexity	0.38	0.40

2.1.4 Gene Prediction and Annotation

For *ab initio* gene prediction, BRAKER1 [38] used 28.26 Gb of RNA-seq data as extrinsic evidence to predict 60,482 genes. For homology-based gene prediction, BRAKER2 [39] generated 57,574 hints and predicted genes. TSEBRA [40] combined BRAKER runs and selected 44,054 best gene predictions. Of these, 66.94% (29,490) were annotated in the Actinopterygii database of UniProtKB [41] or eggNOG [42]; and 33.06% (13,525) were not annotated. We kept the annotated (29,490) and the non-annotated predicted genes with more than 150 amino acids (1,039) for the final dataset, summarizing 30,501 protein-coding predicted genes (**Supplementary Table 3**). The final dataset had 94.1% complete Benchmarking Universal Single-Copy Orthologs (BUSCO), 89% complete and single-copy, 5.1% duplicated, 2.2% fragmented, and 3.7% missing BUSCO. For the functional annotation, we performed blast searches against the Actinopterygii database of UniProtKB [41] and eggNOG [42]. Of all the predicted genes, only 3.34% (1,018) were not annotated.

The most representative gene ontology (GO) terms (> 15% of genes) according to the three-domain can be seen in **Supplementary Figure 2.**

2.2 Linkage map

A total amount of 1,307,500,332 raw reads were sequenced using Double Digest Restriction Site-Associated DNA Sequencing (ddRADseq), resulting approximately 200 Gb of data (about 28 Gb per library). After filtering to remove low-quality sequences and reads with missing or ambiguous barcodes, an average of 11% of the reads were removed from each library, retaining 89% of the reads for analysis. Additionally, 24 individuals were excluded due to a low number of reads (< 1 million). The average number of reads per sample was 4.3 million. The raw sequencing data and filtered reads for each library are detailed in **Supplementary Table 4**.

After mapping the ddRAD reads to the chromosome-level genome, SNP calling analysis identified 41,033 SNPs from 85,167 loci across 281 individuals. Using Plink [43], we applied the mind and geno filters, excluding 56 individuals and 8,971 SNPs. The maf filter further excluded 3,733 SNPs. Consequently, 225 individuals and 28,329 SNPs passed all quality controls (with a total genotyping rate of 0.96) and were used for linkage mapping.

A pedigree test was performed, and individuals with more than 10% Mendelian errors were removed. After calling possible missing or erroneous parental genotypes using the *ParentalCall* module, a total of 9,997 SNPs were grouped into linkage groups (LGs). We calculated several Logarithm of Odds (LOD) scores between markers and selected the best marker distribution based on the species' karyotype characteristics. Although the haploid chromosome number for *M. macrocephalus* is 27, the best marker distribution was achieved using 28 LGs (with LOD 12), likely due to a specific region of the Z chromosome forming a

separate linkage group (**Supplementary Figure 3)**. The remaining markers were assigned to existing LGs using LOD 10, which recovered 1,234 markers. A total of 18,098 markers were discarded because they did not associate with the linkage map. Within each LG, the order of markers with the best likelihood was combined to produce the final linkage map. A total of 11,231 SNPs were assigned to 28 LGs. We constructed male, female, and a sex-averaged maps (average position between male and female maps) (**Figure 3**).

Figure 3. (A) Linkage maps of *Megaleporinus macrocephalus* with 28 linkage groups and 11,231 SNPs. Male and female maps are shown at the top and bottom, respectively, with marker density indicated by a color gradient from blue (low density) to red (high density). (B) Collinearity analysis of linkage groups and chromosomes.

The number of SNPs in the LGs ranged from 710 (LG1) to 203 (LG28). In the sex-averaged map, LGs length varied from 143.08 cM (LG22) to 43.25 cM (LG24), with an average of 3320.36 cM and an average distance between markers of 0.29 cM (SD = 0.12). The highest and lowest marker densities were found in LG1 and LG22, with averages of 0.18 cM and 0.61 cM, respectively (**Supplementary Table 5**).

Regarding sex-specific differences, the average distance between markers in male and female maps were 0.31 cM and 0.29 cM, respectively. Consequently, the male map (3,518.24 cM) was longer than the female (3,301.97 cM). The male-to-female genetic length ratio across the entire genome was 1.07, with ratios varying from 0.54 (LG24) to 1.37 (LG12). The highest recombination density was detected in the proximal region of the centromeres (considering that this species has metacentric/submetacentric chromosomes), although some exceptions occurred in the terminal region of the LG11 (**Figure 3**).

2.2.1 Recombination suppression within LG24

In LG24, recombination was distributed differently between the sexes (heterochiasmy). The female map for LG24 was almost double the size (87.81 cM) compared to the male map (47.37 cM). Collinearity analysis revealed a correspondence between LG24 and sex chromosome 13 (**Figure 3**), particularly in regions < 20 Mb and > 40 Mb. Additionally, zero recombination clusters (areas of recombination suppression) were observed in LG24 for both sexes (**Figure 4**), characterized by blocks of markers that vary in physical distance (bp) but remain consistent in genetic distance (cM). Furthermore, the same chromosome 13 was also associated with LG27, which corresponds to the pseudoautosomal region (PAR) (**Figure 3B**). This suggests that the optimal LOD value resulted in 28 linkage groups (n = 27 chromosomes), as both LG24 and LG27 correspond to different regions of the same sex chromosome. It is important to note that a small part of LG24 also showed collinearity with autosomes (chr 25 and chr 27).

Figure 4. Position of markers in the genome (in Mb) versus their position on the genetic map (in cM) for LG24. The female map is shown at the top, and the male map at the bottom. Recombination varies on the physical map but not on the genetic map, forming vertical structures of clusters known as zero recombination clusters, which are highlighted by circles.

2.2.2 Discordance between physical and genetic mapping

To integrate the genome assembly with the linkage map data, we ordered the genome scaffolds using the linkage map as a reference. Chromonomer [44] attempts to identify and remove markers that are out of order in the genetic map when considered against their local assembly order and to identify scaffolds that have been incorrectly assembled according to the genetic map, splitting those scaffolds as necessary. This ordering grouped 1,575 map markers into 352 scaffolds. The remaining SNP loci were not used for genome anchoring because they

were not aligned to the piauçu scaffolds or were markers mapping to multiple regions, or loci where the orientation could not be suitably assigned. These results allowed the construction of a chromonome that clustered 75% (1,221,855,406 bp) of the initial scaffold data into 27 pseudomolecules (chromosomes) totalizing 977 Mb in length. However, 320 Mb were not anchored in pseudomolecules. LG24 anchored a low number of scaffolds, resulting in a pseudomolecule with poor scaffolding and small size (~ 4 Mb). This can be explained by the region of sex conflict between the Z and W chromosomes and the consequent suppression of recombination between them.

The dot plot synteny analysis demonstrated a high degree of concordance between the chromosomes scaffolded with Hi-C data (physical mapping) and those scaffolded with the genetic map (chromonome) (**Supplementary Figure 4**, illustrated by chromosomes 5, 8, and 20). Insertions and deletions were observed in all chromosomes (*e.g.*, chromosome 2, where 25% of the initial scaffold data was missing). Additionally, structural differences were noted in some chromosomes, revealing relocations (chromosomes 1 and 21) and major inversions (chromosomes 3, 18, and 19).

2.3 Highly differentiated regions in ZW chromosome

Whole-genome resequencing of male and female pools yielded 266,697,484 and 231,722,384 paired-end clean reads, respectively. These reads were then mapped to the female chromosome-level genome to identify genomic regions enriched for sex-biased signals, such as differences in coverage between males and females or sex-biased SNPs. The mapping rates of paired-end reads from the male and female pools were 98.74% and 97.08%, respectively, with average depths of 25x for the male pool and 24x for the female pool.

The SNP distribution analysis revealed a strong sex-linked signal in females and high F_{ST} values in the sex chromosome (chromosome 13) (Figure 5). This profile supports the presence of a female heterogametic system (ZW/ZZ), as previously reported by cytogenetic data [7].

Figure 5. Plots of F_{ST}, female-specific SNPs, and male-specific SNPs across the 27 chromosomes of the *Megaleporinus macrocephalus* genome. Higher F_{ST} values and the presence of sex-specific SNPs in chromosome 13 confirm the ZW sex determination system.

2.3.1 Distinct patterns in the sex chromosome

The sex chromosome, which is approximately 45 Mb in length, was divided into three regions according to the overall characteristics of read depth (coverage), the pattern of sex-specific SNPs, and F_{ST}. The first region comprises the beginning of the chromosome, from 0 to approximately 3 Mb, and is characterized by high coverage in females (2.3-fold the female average depth), and low coverage in males (0.6-fold the male average depth, **Figure 6**). The absence of coverage in males was detected in some areas (depth ratio ≈ 0). In addition, the major F_{ST} peak (F_{ST} = 0.17) was located within this region (**Figure 6**). The observed patterns strongly suggest the assembly of W-specific sequences in this region. This region was named a putative W-region (PWR), characterized by high differentiation, as confirmed by the absence of recombination in the linkage map (**Figure 4**).

Figure 6. The sex chromosome of *Megaleporinus macrocephalus*, divided into three major regions: putative W-region (PWR), from 0 to 3 Mb, chimera (CHR), from 3 to 20 Mb and 44 to 45 Mb, and pseudoautosomic region (PAR), from 20 to 44 Mb. (A) Distribution of sex-specific SNPs, with red indicating female-specific SNPs and blue indicating male-specific

SNPs. (B) Male-to-female (M/F) depth ratio (absolute depth of males/absolute depth of females). A depth ratio of 1 indicates equal read coverage in both sexes; a depth ratio > 1 indicates higher coverage in males and a depth ratio < 1 indicates higher coverage in females. (C) Fixation index (F_{ST}) values, highlighting regions of high differentiation.

The second region encompasses approximately 3 to 20 Mb, reaching the opposing terminal segment of the sex chromosome, which ranges from ~44 to 45 Mb (**Figure 6**). Within this zone, two distinct patterns were recognized. The first, prevalent in most of the region, exhibited Z-specific characteristics, as males demonstrated at least double the coverage of females (*i.e.*, males have two copies of Z, while females have one). The other pattern was characterized by a high density of female-specific SNPs, with peaks summarizing more than 2,000 SNPs, representing allelic differences between Z and W sequences. It is important to note that the SNPs identified in the female pool may also include those specifically from the Z chromosome. However, these are likely in low quantity, as the male pool showed a low number of Z-specific SNPs, with an average of 13 SNPs per kilobase. This low quantity likely does not affect the demarcation of the region of sex conflict.

Furthermore, in the areas where W sequences were observed, males had no coverage, and females had 2.6-fold the average depth, resulting in a depth ratio of approximately 0 (**Figure 6**). This observation was supported by a higher recombination frequency in males compared to females within this region of LG24 (3 to 20 Mb and 44 to 45 Mb, as illustrated in **Figure 4**). This evidence indicates a certain degree of similarity between the Z and W sequences, allowing them to be scaffolded in the same region. Therefore, this locus was named “chimera” (CHR), which is undergoing degeneration, as indicated by differences between females and males in recombination suppression, coverage, F*st*, and number of SNPs.

The region comprising approximately 20 to 44 Mb was characterized by a lack of sex-specific SNPs (**Figure 6**). In this genomic locus, an almost equal absolute depth between males and females was also observed (depth ratio ≈ 1, **Figure 6**). This illustrates homology between the male and female sequences in this zone and could indicate normal recombination rates, as seen in pseudo-autosomal regions. Therefore, we named this region the PAR.

2.4 Differential expression between males and females

A total of 28.26 Gb of gonadal paired-end RNA-seq data was pseudo-aligned with 30,500 transcripts of *M. macrocephalus* (**Supplementary Table 6**). Approximately 99.9% (30,460) of the RNA-seq transcripts were successfully pseudo-aligned. After filtering out low counts (≤ 1), 27,120 transcripts remained for differential expression analysis. Principal component analysis (PCA) revealed that Principal Component 1 (PC1) accounted for 78% of the variance in the data. As expected, the samples clustered into two groups along PC1: ZZ males and ZW females, with minor intra-variation observed within the male group (**Figure 7A**). This clustering pattern was corroborated by the heatmap of the Euclidean distance matrix (**Figure 7B**).

Figure 7. Clustering of RNA-Seq gonadal samples of *Megaleporinus macrocephalus* by transcript expression. (A) PCA plot. (B) Heatmap of the Euclidean distance matrix. (C) Volcano plot indicating down-regulated (left, red) and up-regulated (right, red) transcripts. Non-significant transcripts (*p*adj > 0.01) are shown in black.

The analysis identified 2,557 differentially expressed transcripts (*p*adj ≤ 0.01). Of these, 42.66% (1,091) were up-regulated in males and 57.33% (1,466) in females (**Table 3 and**

Figure 7C). Most of these differentially expressed (DE) transcripts were components of the zona pellucida and were up-regulated in females (**Supplementary Table 7**).

Within the sex chromosome, males exhibited a higher number of up-regulated and DE genes compared to the average per chromosome. The putative W-region (PWR) had the highest concentration of DE genes. Additionally, males showed significantly more up-regulation in the chimeric region (CHR) than females, a trend also observed in both the PWR and the pseudoautosomal region (PAR) (**Table 3A**).

Table 3. Differentially expressed genes in gonads of males (ZZ) and females (ZW) of *Megaleporinus macrocephalus* during sex determination. (A) Comparison of differentially expressed genes between autosomes and the sex chromosome. (B) Distribution, expression, and differential expression of genes in sex chromosome regions. Regions in the sex chromosome: putative W-region (PWR), chimera (CHR) and pseudoautosomal region (PAR).

A.

**males ZZ vs females ZW**	**autosomes**		**sex chromosome regions**			
	total	average/ chr	PWR	CHR	PAR	total
differentially expressed[1]	2,557	95	12	82	54	148
male up-regulated[2]	1,091	40	5	62	26	93
female up-regulated[3]	1,466	54	8	21	29	58

[1] *p*adj ≤ 0.01.
[2] LFC ≥ 1, *p*adj ≤ 0.01.
[3] LFC ≤ -1, *p*adj ≤ 0.01.

B.

	**size (Mb)**	**no. of genes**	**E[1]**	**% E[2]**	**% DE[3]**
PWR	3	75	50	66.67	24.00
CHR	18	465	359	77.20	22.84
PAR	24	472	442	93.64	12.22

[1] number of expressed genes.
[2] percentage of expressed genes (count > 1).
[3] percentage of expressed genes that were differentially expressed.

The PWR displayed the lowest percentage of expressed genes, suggesting potential functional loss in some genes. Despite its size of only 3 Mb, 24% of its expressed genes were DE (**Table 3B**). In contrast, the PAR, which spans 24 Mb, had only 12.22% of DE genes. The lower expression in the PWR and CHR compared to the PAR indicates a degree of degeneration in these regions (**Table 3B**).

The sex chromosome harbored several genes related to sex determination and differentiation, as listed in **Table 4**. None of the candidate sex genes have paralogs on autosomes, simplifying their analysis and interpretation. The pseudoautosomal region (PAR) contains the largest number of sex candidate genes, with five genes, followed by the chimera region (CHR) and the putative W-region (PWR), with four and one gene, respectively. Except for *igfbp6b*, all genes in the CHR exhibit a high density of female-specific SNPs (gene average density > 500 SNPs), with *wnt4* showing the highest density. These genes supposedly contain sequences on both the Z and W chromosomes. The Z sequences align regularly with the reference genome, whereas the W sequences, although homologous to the reference genome, display allelic differences seen as SNPs. In contrast, all candidate genes present a low density of male-specific SNPs. *Igfbp6b*, *wnt4*, *sox12*, *foxp4*, *amhr2*, and *wnt7a* are up-regulated in males, while *ccdc114*, *ccdc71*, *sox13*, and *bmp7* are up-regulated in females. Notably, *igfbp6b* and *amhr2* are differentially expressed and up-regulated in males.

Since the Z and W chromosomes are not phased, we were unable to perform in-depth genomic analyses of the evolutionary processes of sex chromosomes, such as the detailed structure of genes in the non-recombining region. This limitation prevents the estimation of sequence divergence estimates in coding sequences or even the gene loss rate. Additionally, the lack of genomic resources from closely related species also prevents comparative synteny

analysis. The closest related species with available genomic resources belong to the family Serrasalmidae, which diverged from Anostomidae approximately 70 million years ago [45].

Table 4. Sex determination-related genes identified on the *Megaleporinus macrocephalus* sex chromosome, including their location, average density of the gene female-specific SNPs (F-SNPs) and male-specific SNPs (M-SNPs), log fold change (LFC), and *p*-adjusted values. Regions in the sex chromosome: putative W-region (PWR), chimera (CHR) and pseudoautosomal region (PAR).

gene ID	description	region	F-SNPs	M-SNPs	LFC*	*p*adj
ccdc114	Coiled-coil domain containing 114	PWR	1	2	-5.50	1
*igfbp6b***	Insulin-like growth factor binding protein	CHR	4	2	5.19	$3.48e^{-13}$
ccdc71	Coiled-coil domain containing 71	CHR	519	6	-1.25	1
wnt4	Wingless-type MMTV integration site family, member 4	CHR	1,663	17	1.57	1
sox13	SRY (sex determining region Y)-box 13	CHR	1,426	1	-0.50	1
sox12	SRY (sex determining region Y)-box 12	PAR	9	10	3.45	$9.80e^{-2}$
foxp4	Forkhead box	PAR	14	23	1.54	1
bmp7	Bone morphogenetic protein	PAR	14	15	-1.67	$6.88e^{-1}$
*amhr2***	Anti-Mullerian hormone receptor type II	PAR	1	0	3.80	$1.40e^{-6}$
wnt7a	Wingless-type MMTV integration site family, member 7A	PAR	105	16	3.71	$1.73e^{-1}$

* If LFC ≥ 1, up-regulated in males, if LFC ≤ -1, up-regulated in females.
** means differentially expressed genes (*p*adj ≤ 0.01).

3. Discussion

3.1 Chromosome-level genome

In this study, we employed multiple genome sequencing strategies to assemble a chromosome-level reference genome for *Megaleporinus macrocephalus*, a Neotropical fish species with heteromorphic sex chromosomes. Despite the significant morphological and ecological diversity within Characiformes [45], genomic data for this order remain scarce.

The piauçu genome demonstrated quality, contiguity metrics (contig and scaffold N50), and size comparable to other available assemblies of Neotropical fish (**Supplementary Table 8**). The repeat content found in the piauçu genome (46.71%) was intermediate compared to other Neotropical fish species, such as *C. macropomum* (52.49%) [46] and *A. mexicanus* (41%) [47]. DNA transposons were the most abundant type of transposable elements, accounting for 11.82% of the genome, consistent with observations in other teleost fish [48]. A substantial portion (18.89%) of the interspersed repeats remained unclassified, similar to findings in tambaqui [46] and red-bellied piranha [49] genomes, which reported 39.15% and 28.3% unclassified sequences, respectively. Overall, the repeat content in the sex chromosome was slightly higher than in the autosomes (4.24%), which is expected. In the repeat landscape (**Supplementary Figure 1**), two bursts of transposition dominated by DNA transposons were observed, similar to what has been reported in other teleost fish, such as Nile tilapia [50].

Genome annotation identified 30,501 predicted protein-coding genes **(Supplementary Table 3)**, which is consistent with other related Neotropical fish genomes, such as tambaqui [46], red-bellied piranha [49] and cavefish [51] (31,149; 30,575 and 25,293, respectively).

3.2 Satellite DNA

Satellite DNAs (satDNAs) are composed of arrays with nearly identical repeating units. These units can range in length from single base pairs (mononucleotide repeats) to several megabases without interruption [52]. The considerable length of these arrays presents a challenge for modern sequencing, assembly, and mapping techniques, making the analysis of long fragments problematic [53]. Long reads are more capable of characterizing the variable satellite content and assembling difficult, repetitive parts of the genome [54].

Piauçu is noted for having the highest number of characterized satellites for any species so far [12]. However, even using the species' satelitome in the repeat annotation, the amount of satDNA found in our assembly was significantly lower than expected. This suggests that:

1. The long reads were not capable of capturing the complete arrays of these repeats. High-identity regions, such as tandem repeats, often collapse during assembly with short or erroneous long reads [54] [55].
2. The pipelines used to annotate the repeats were not effective in identifying the satellite DNA arrays. According to [56], computational tools that account for the high error rates of long-read technologies are lacking. Using personalized pipelines, like those in [29] [57], and [58], or tools specifically designed for satellite analysis in long reads, such as NCRF [56], tandem-genotypes [59], P ACMON STR [60], TandemTools [61] and Winnowmap2 [62], could improve the satellite DNA annotation results.
3. SatDNAs may have been removed during the scaffolding process. Conventional Hi-C analysis often fails to account for reads that map to multiple locations, underestimating biological signals from repetitive genome regions [54] [58]. This disproportionately affects repetitive parts of the genome, such as sex chromosomes [54] [29] [58] [63].

3.3 Linkage map

In this study, we achieved a resolution similar to other linkage maps constructed for related Neotropical fish species with analogous karyotype characteristics (haploid chromosome number, morphology, and size) (**Supplementary Table 9**). The sex chromosome showed collinearity with two linkage groups, LG24 and LG27, which represent the Z-recombining region and PAR of the sex chromosome, respectively. This pattern was previously reported in the butterfly *Melitaea cinxia* [64], which also has a ZW sex chromosome system. LG24 consisted of markers that followed a Z chromosomal inheritance pattern, where female offspring are homozygous for one of the father's alleles. This explains the strong heterochiasmy observed in this LG, with higher recombination in the male map. Although LG27 was separated by the LG24 linkage pattern, it is physically merged with LG24 in the genome and corresponds to the pseudo-autosomal region of the sex chromosome, as similarly observed in *M. cinxia* [64].

The linkage map for male piauçu was longer than that for females, with a genetic length ratio of 1.07. Recently, the tambaqui *C. macropomum* was described as having a hypothetical XY sex determination system [65], despite not presenting heteromorphic sex chromosomes. In contrast, [66] found that the female linkage map in tambaqui was larger than the male map (1.55x). Differences in map length can result from variations in the number of recombination events in the two parents, as well as differences in the number and location of the mapped loci. It is common to find differences in recombination ratios between the sexes in most aquatic species [67] [68] [69] [70]. Despite this being a common phenomenon, the mechanism responsible for different recombination rates between the sexes is still not well understood [71]. This explains the opposite sex-specific differences observed between piauçu and tambaqui and suggests that the heterogametic sex tends to have smaller maps due to recombination suppression [70].

The influence of the sex determination system on sex-specific recombination patterns has also been described for other fish lineages. In flatfish species such as turbot [72], Senegalese sole [73], and Atlantic halibut [70], female maps were larger (1.36, 1.32, and 1.07 times, respectively). Conversely, in Japanese flounder *Paralichthys olivaceus* [69] and tongue sole *Cynoglossus semilaevis* [71], the male maps were slightly larger (1.03 and 1.09 times, respectively).

3.3.1 Inconsistencies between genetic and physical mapping

Our linkage map was successfully used as a reference to anchor the genome scaffolds into a chromosome-level assembly, underscoring its high quality. The chromosome-level genome anchored using the linkage map, showed a high correspondence with the reference genome scaffolded using Hi-C physical mapping. These results are consistent with those obtained in other chromosome-level genomes anchored with linkage maps, such as in *A. mexicanus* [51] and *Sander lucioperca* [74]. The inconsistencies revealed by structural differences, such as relocations and inversions, were also reported in the Lake Trout *Salvelinus namaycush* [75]. These inconsistencies will likely require further investigation using additional techniques, such as physical mapping of specific DNA sequences onto chromosome spreads with fluorescent in situ hybridization (FISH).

3.4 Sex chromosome characterization

Due to the complexity of sex-determining regions (SDRs) and the reduced sequencing coverage in XY or ZW genotypes compared to autosomes, assembling sex chromosomes is significantly more challenging than assembling autosomes [76]. As a result, sex chromosomes are often the least well-assembled and annotated regions [26]. Accurate phased assembly of

sex chromosomes requires an additional analysis process to separate the contigs (Z or W reads) belonging to the non-recombining region of the sex chromosomes [76]. Therefore, fish sex chromosome assemblies generally rely in a previously established genome of the homogametic sex, which contains the sex chromosome, as a reference to identify the sex-linked sequences [27]. In this case, an initial assembly is performed with long reads, and the resulting contigs are aligned to the previously stablished reference genome and sex chromosome. Subsequently, based on empirically established thresholds for parameters such as alignment percentage and similarity, the contigs linked to the W/Y and Z/X chromosomes are identified. For example, in the assembly of the stickleback Y chromosome [29], the putatively Y-linked contigs were identified as those that aligned only partially (< 25% of the contig length) or did not align at all to the female reference genome, or those that aligned to the reference X chromosome (> 25% of the contig length) but with greater sequence divergence. Conversely, the putatively X-linked contigs were identified as those that aligned to the reference X chromosome (> 25% of the contig length) but with lower sequence divergence (greater than 96% similarity). After selecting the contigs linked to each sex chromosome, they can be ordered and grouped separately using scaffolding techniques [29] or the long reads associated with these contigs can be extracted and assembled separately using stricter assembly parameters [77].

An alternative approach to separately assembling the Z and W chromosomes involves leveraging information from the linkage groups. Since LG24 contains markers consistent with a Z-chromosomal inheritance pattern, where female offspring are homozygous for one of the father's alleles, it is theoretically possible to isolate Z reads for further assembly. However, in our study, we concluded that this approach would still likely result in spurious assemblies of the Z and W chromosomes. The primary issue is the limited number of markers in LG24 (only 225, as shown in **Supplementary Table 5**), which is insufficient to robustly support the Z chromosome assembly. This limitation resulted in a relatively small chromosome (~4 Mb),

after scaffolding with Chromonomer [44] (**Supplementary Table 5**), whereas a chromosome region of approximately 17 Mb had been expected.

In the absence of a reference, a *k*-mer approach, as used in *Verasper variegatus* [33], could be applied to assemble the sex chromosomes separately. This method uses resequencing data from both males and females to identify female-specific *k*-mers. Once these *k*-mers are identified, female-specific data are filtered from the PacBio HiFi and Hi-C sequencing datasets. The HiFi data are then used to assemble candidate W sequences, which are subsequently anchored to chromosomes using the female-specific Hi-C data. While this approach has proven effective, it requires highly accurate long reads, such as HiFi data, which were unavailable for our study.

Despite significant advances in genome sequencing and assembling techniques, a major challenge persists because genome assembly algorithms are not typically optimized for sex chromosomes [76]. In this study, we faced this issue, as accurate and phased assembly of the Z and W chromosomes was not possible. Current PacBio HiFi assembly algorithms are created to phase structurally similar autosomes into distinct allelic haplotypes. However, sex chromosomes often deviate from this pattern due to their potential heteromorphy, which can involve differences in size, gene content, repeat content, and structural variation between the sex chromosome pairs [76]. For instance, the human Y chromosome has been notoriously challenging to assemble due to its complex repetitive structure [78], resulting in more than 50% of the chromosome missing from the last reference assembly [79]. Only recently, the Telomere-to-Telomere (T2T) consortium published the complete and gapless sequence of the human Y chromosome [80], using an innovative methodology [80] [81] [82]. Briefly, the strategy utilizes assembly string graphs constructed using PacBio HiFi reads, ONT ultra-long reads for resolution of repeats and sub-graphs, trio-binned data to patch coverage gaps caused by HiFi sequencing biases, and extensive manual curation. The assembler released with the

development of the T2T methodology has enabled the complete assembly of several ape X and Y chromosomes [83][84].

In our study, we assembled a highly degenerated sex chromosome in a non-model species without prior genomic references, utilizing PacBio long reads and Hi-C. The absence of an initial Z chromosome reference, coupled with the use of error-prone PacBio CLR reads, prevented the accurate separation of homologous sequences of Z and W in the non-recombining region of the sex chromosome, as also observed in *V. variegatus* [33]. Despite the consensus sex chromosome, the integration of different approaches (recombination suppression, coverage, F_{st}, and number of SNPs) allowed the identification of 3 Mb of a highly differentiated putative W-specific region, 18 Mb of a region undergoing degeneration, and 24 Mb of the PAR.

3.5 Sex chromosome gene repertoire

Genes situated in the non-recombining region, namely *ccdc114, igfbp6, sox13,* and *wnt4,* belong to gene families involved in various developmental processes in fish, including sex differentiation and determination [85]. Despite the relevance of their gene families in sex determination/differentiation, neither *ccdc114, igfbp6* nor *sox13* are currently recognized to actively participate in these processes or have been identified as master sex-determining (MSD) genes. *Wnt4* plays a crucial role in ovarian differentiation and development in mammals. However, the role of *wnt4* in teleost fish; remains unclear [86]. In tambaqui (*C. macropomum*), it was related to sex differentiation, either upregulated in female-like individuals or antagonized in male-like individuals [60], suggesting that it could also play a role in sex regulation and dimorphism in piauçu.

Within the recombining region, genes from the TGF-β signaling pathway, such as *bmp7* and *amhr2,* were identified. Members of this signaling pathway have recurrently and

independently emerged as master sex-determining (MSD) genes [87]. Notably, of the 20 distinct MSD genes identified so far, 13 belong to the TGF-β signaling pathway, including *amh, amhr2, bmpr1b, gsdf,* and *gdf6*.

Bone morphogenetic proteins (BMPs) are implicated in mammalian germ cell specification and gametogenesis [88]. Recently, a truncated form of a BMP type I receptor, BMPR1BB, was identified as the MSD gene in *Atlantic herring* [30]. While *bmp7* has not been identified as a candidate sex-determining gene in any species thus far, it has been linked to sex differentiation processes in mouse embryos [89] and fish [90], requiring further studies to understand its role in sex determination in piauçu. *Amhr2*, the anti-Müllerian hormone receptor, has been co-opted as an MSD gene in some fish species [91] [92] [93]. Due to the relevance of the *amh*/*amhr2* pathway in sex determination, especially in fish, we highlight *amhr2* as another candidate for sex determination in piauçu. We hypothesize that a long-distance receptor located in the non-recombining region is inhibiting *amhr2* transcription, directing the sex fate toward females. A similar mechanism was reported in the Amami spine rat [94].

4. Methods

4.1 Chromosome-level genome

Tissue samples for genome sequencing were obtained from an adult ZW female *Megaleporinus macrocephalus* from the broodstock of the Aquaculture Center of São Paulo State University. To confirm the genotype of the individual, we performed cytogenetic analysis using the lymphocyte culture technique described by [95] with some adjustments, and C-banding according to Sumner, 1972 (**Supplementary Figure 5**).

High molecular weight (HMW) DNA was extracted from blood using the Nanobind CBB Big DNA Kit (Circulomics) to generate long reads, and a continuous long read (CLR) library

was constructed using the SMRTbell Express Template Prep Kit 2.0. The library was sequenced in one single-molecule real-time (SMRT) cell on the PacBio Sequel II System (RRID:SCR_017990). All these steps were performed by the Genomics & Cell Characterization Core Facility (GC3F) of the University of Oregon (USA).

To improve the accuracy of the long reads, a short read library was produced using the MGIEasy PCR-Free Library Prep Set (MGI Tech Co., Ltd.) and sequenced on a BGI DNBSEQ-G400 (RRID:SCR_017980) 150 bp paired-end reads at the BGI Genomics facility. Subsequently, to merge the scaffolds into putative chromosomes, a chromatin interaction (Hi-C) library was generated using the Proximo Hi-C Library Prep Kit (Phase Genomics) with *in vivo* cross-linking at the Genomic Sciences Laboratory of the North Carolina State University (USA). Sequencing was performed on an Illumina NovaSeq 6000 (RRID:SCR_016387)150 bp paired-end reads.

4.1.1 Genome Size Estimate

The short reads were used to estimate the haploid genome size, rate of heterozygosity, and abundance of repetitive elements. First, the reads were trimmed with Trimommatic [97] to remove bases with an average quality of less than 20 within a sliding window of 4 bp and bases with quality less than 20 at the beginning and the end of the reads. Reads shorter than 36 bp were also discarded. After filtering, Jellyfish (RRID:SCR_005491) [98] was used to count canonical *k*-mers (-C flag) of lengths ranging from 21 to 24. The resulting *k*-mer profile was then loaded into GenomeScope (RRID:SCR_017014) [99] for analysis.

4.1.2 Genome Assembly

We used Falcon (RRID:SCR_023199) / Falcon-Unzip [34], Flye (RRID:SCR_017016) [100], wtdbg2 (RRID:SCR_017225)[101], and Canu (RRID:SCR_015880) [102] to assemble

the PacBio long reads. The Falcon/ Falcon-Unzip [34] assembly presented the best contiguity metrics and was chosen for further analysis. The initial contig assembly was performed using a minimum read length cutoff of 5,000 bp. Falcon [34] was run with default parameters, except for computing the overlaps. Raw read overlaps were computed with the following daligner parameters: -v -k16 -w7 -h64 -e0.70 -s1000 -M27 -H5000, to better reflect the higher error rate in PacBio Sequel II reads. Preassembled read (pread) overlaps were computed with daligner parameters: -v -k20 -w6 -h256 -e0.96 -s1000 -l2500 -M27 -H5000. Falcon-Unzip [34] was run with default parameters and resulted in a set of primary and alternate contigs. False duplications in the contigs were removed using Purge_Dups (RRID:SCR_021173). Short-read polishing was performed with Polca [103]. To polish the primary and alternate assemblies, we first concatenated them and then followed with one round of short-read polishing. To improve the assembly's contiguity, PacBio long reads > 10 kb were used to fill in spanned gaps with SAMBA (RRID:SCR_006557) [104]. The Juicer and 3D de novo assembly (RRID:SCR_017227) pipelines [105] were used to orient scaffolds into putative chromosomes. First, a file with the location of DpnII enzyme restriction sites in the assembly was generated (*generate_site_positions.py*) along with a file containing scaffold sizes. Second, Hi-C reads were aligned to the assembly and filtered by Juicer (RRID:SCR_017226) [106] to generate a duplicated-free list of paired alignments (merged_nodups file). Finally, 3d-dna [105] was run with a minimum scaffold size of 10 kb. The resulting contact map was manually curated in Juicebox Assembly Tools (JBAT) (RRID:SCR_021172) following a post-curation process. An additional round of polishing was then performed with Polca [103].

4.1.3 Quality Assessment of Genome

The correctness of the genome assembly was evaluated at each assembly step using Merqury (RRID:SCR_022964) [36]. This tool compared assembly *k*-mers to those found in the

unassembled, highly accurate MGISEQ short reads to estimate base-level accuracy (consensus quality value, QV) and *k*-mer completeness. The QV represents a log-scaled probability of error for the consensus base calls. Contiguity measures, such as contig and scaffold N50, were obtained using the *stats.sh* script of BBMap (RRID:SCR_016965). To assess the completeness of the genome, we performed a BUSCO analysis (RRID:SCR_015008) [107] using the Actinopterygii dataset. Additionally, the assembly was verified for contamination following the National Center for Biotechnology Information (NCBI) submission protocols. Any contaminated scaffolds identified were removed.

4.1.4 Karyotype Validation

To validate the quality of our assembly, we performed a Pearson's correlation analysis comparing the estimated size in base pair (bp), based on the average karyotype size in micrometers (µm), with the assembled size (bp) of each chromosome. We measured both arms of each chromosome pair in the female karyotype and calculated an average size (µm) for each chromosome. The estimated chromosome size was then calculated using the formula: chromosome average size (µm) x total genome size (bp) / total karyotype size (µm).

4.1.5 Repeat Annotation

We used RepeatModeler2 [108], with the LTR option enabled, to produce a custom *de novo* library of the repeats present in the genome. Next, Repeat Masker was used to identify, classify, and mask repetitive elements, including low-complexity sequences and interspersed repeats. A combined library was used to run Repeat Masker. First, the RepBase RepeatMasker Edition (version 20181026) was combined with the Dfam library using the *addRepBase.pl* and *configure.pl* scripts. Then, only the repeats present in Teleost were selected using *famdb.py*.

Finally, the custom de novo library, the Teleost repeat sequences, and a satellite library of the species [12] were concatenated.

4.1.6 Gene Prediction and Annotation

We performed gene prediction using de novo, transcriptome and homology-based methods via the BRAKER [38] pipeline. Initially, BRAKER1 [38] utilized RNA-seq data as extrinsic evidence to predict introns and exons boundaries. Subsequently, BRAKER2 [38] incorporated protein homology information from Orthodb sequences of Vertebrata (odb10_vertebrata) [109]. Finally, TSEBRA [40] was employed to select the best annotations from both predictions, enhancing the accuracy of the gene models.

To assign functional annotation to the gene models, we performed searches using the predicted proteins with the Actinopterygii dataset of UniProtKB [41] and EggNOG-mapper [110] [42]. The search results were loaded into Blast2GO [111], mapped, and annotated. We further conducted a sanity check on the dataset to include only high-quality predictions. All predicted protein-coding regions with no functional assignment and showing less than 150 amino acids in length were not considered high-quality predictions. The quality of the annotation was evaluated using BUSCO [112].

4.2 Linkage mapping

To construct a linkage mapping, we produced four full-sib families using single mating (1 female x 1 male) during the breeding season of December 2018, totaling 299 progeny individuals (**Supplementary Table 10**). The breeders belonged to the population maintained at the Aquaculture Center of São Paulo State University (UNESP), Jaboticabal (São Paulo State, Brazil).

Induced spawning was performed using carp pituitary extract dissolved in saline solution (0.9% NaCl), applied in two dosages with a 12-hour interval: the first and second doses were 0.6 and 5.4 mg/kg for females, and a single dosage of 1.5 mg/kg for males, administered at the same time as the females' second dose. After hatching in 20 L conical fiberglass incubators, the larvae were transferred to tanks of 250 L. The larvae were fed with *Artemia nauplii* for 20 days. Gradually, the feed was replaced with a diet containing 50% crude protein. At the fingerling stage, they were fed with 1.2 mm pelleted feeds containing 40% crude protein, provided twice daily (commercial feed Nutripiscis Presence).

Each full-sib family was kept separately in individual 1 m3 fiberglass tanks until they were 6 months old. The fish were maintained in a water recirculation system equipped with mechanical and biological filters, an external aeration system, and a temperature control system set to 30 °C (standard deviation = 0.5 °C) using a thermal controller connected to two 500-watt heaters. Temperature, dissolved oxygen, and pH were measured using a Multiparameter Water Quality Checker U-50 (Horiba).

After this period, we collected blood samples for genomic analyses and recorded the weight of all animals using an analytical balance (average weight was 6 g). The fish were then euthanized for sex identification. Individual sex was verified by a PCR-based protocol using a chromosome W-probe [12] as well as by cytogenetic analysis. Chromosome preparations were obtained from kidney tissues using the technique described by [113].

4.2.1 SNP genotyping

DNA was extracted from blood samples using the Wizard Genomic DNA Purification kit (Promega), and quality was verified through 1% agarose gel electrophoresis. Purity was accessed with a Nanodrop One, and concentration (ng/µl) was measured using a Qubit fluorometer with the Qubit dsDNA HS Assay kit (Invitrogen, USA). We used a modified

version of the protocol described by [114] to construct ddRADseq libraries. Briefly, 75 ng of genomic DNA from each individual was digested (8 U/reaction) using a combination of two restriction enzymes, SphI and MluCI (New England Biolabs), and ligated to specific adapters (P1 and P2, 0.25 μM) using T4 DNA ligase at 23°C for one and a half hours, followed by 65°C for 10 minutes to inactivate the enzyme. The P1 adapters included an additional 5 nucleotides serving as individual tags (barcode). The selection of digested fragments was performed using E-Gel Power Snap System (Thermo Fisher Scientific), targeting fragments of approximately 350 bp. Subsequently, PCR assays were performed to incorporate the identification of each library. In total, 7 libraries were constructed, with an average of 46 samples per library. PCR was performed using the Platinum SuperFi DNA Polymerase enzyme (Thermo Fischer Scientific). The reactions were purified with the ProNex Size-Selective Purification System kit (Promega) and the concentration was rechecked by fluorometry using the Qubit 3.0 instrument (Thermo Fisher Scientific). Finally, the libraries were sequenced in 2 lanes of Illumina Hiseq2500 150 PE, using 15 % PhiX (Novogene).

The overall quality of raw sequencing data was checked using FastQC. Next, the data were analyzed using Stacks [115] for SNP calling. Briefly, sequences were demultiplexed and filtered using *process_radtags* and individual reads that passed the previous quality filters were aligned to the chromosome-level reference genome of *M. macrocephalus*. Subsequently, *gstacks* created loci by incorporating the ddRAD-aligned reads. Finally, *populations* was used to generate genotype data for the samples. To differentiate putative SNPs from sequencing errors, we used Plink 1.9 [43] to filter spurious SNPs with more than 10% genotyping error rate (--geno 0.1), minor allele frequencies less than 0.05 (--min-maf 0.05), and Hardy-Weinberg imbalance (p < 5E10-5). Regarding the removal of individuals, samples that had more than 15% (--mind 0.15) of missing genotypes were excluded.

4.2.2 Linkage map

A linkage map was created using Lep-MAP3 [116]. First, a parenthood test was performed using the *IBD* module, and individuals with more than 10% of Mendelian errors were removed. The *ParentCall2* module was used to impute possible missing genotypes or correct erroneous parental genotypes based on progeny data. The *Filtering2* module was used to remove markers with significant segregation distortion (dataTolerance = 0.001) and non-informative markers. Markers were assigned to LG using the *SeparateChromosomes2* module with the minimum LOD score. The best LOD score was selected iteratively, ensuring marker distribution across the first 27 linkage groups, corresponding to the haploid chromosome number of the species. Next, orphan markers were assigned to existing linkage groups (using a lower LOD score than in *SeparateChromosomes2*) using the *JoinSingles2* module and ordered within each linkage group using the *OrderMarkers2* module. Due to the slight stochastic variation in marker distances between runs, the *OrderMarkers2* module was run 15 times, and the order with the best likelihood value for each LG was selected.

The reliability of the SNP *loci* attribution to the LGs and the respective *loci* ordering within the LGs was verified through comparative genomic collinearity analysis with the reference genome using *Circa*.

We used the genome scaffolds to generate another chromosome-level genome using the linkage map as a reference in Chromonomer [44]. This was done to identify possible differences between the linkage map ordering (genetic mapping) and the Hi-C ordering (physical mapping). Chromonomer [44] attempts to find the best set of non-conflicting markers that maximizes the number of scaffolds in the resulting genome while minimizing ordering discrepancies. This process resulted in a FASTA file (chromonome.fa), the chromosome-level genome oriented according to the genetic map.

4.3 Resequencing (pool-sequencing)

We used resequencing analyses to contrast whole-genome sex differences in *M. macrocephalus*. For this purpose, we collected samples from 20 males and 20 females originating from four commercial fish farms in Brazil. Briefly, fish were anesthetized with 0.1% benzocaine for blood collection. The sex of each fish was verified by cytogenetic analysis, as detailed in section **4.2 Linkage mapping**, and samples were clustered into separate male and female pools.

DNA was extracted individually and quantified as described in section **4.2 Linkage mapping**, and then clustered into male and female pools. Library construction and sequencing were performed at INRAE (Rennes, France) in the Laboratory of Physiology and Genomics of Fish (LPGP) using an Illumina NovaSeq S4 platform with 150 bp paired-end reads.

The Pool-Seq dataset was analyzed using the Pooled Sequencing Analysis for Sex Signal (PSASS) pipeline. Briefly, reads from the male and female pools were mapped to the female pseudo-haplotype chromosome-level genome (GCA_021613375.1) using bwa-mem [117] with default parameters. The alignment files were then sorted, merged and PCR duplicates were removed using Picard tools. Reads with mapping quality < 20 and those not uniquely mapped were also removed using samtools [118]. Next, the two sex BAM files were used to generate a pileup file with samtools mpileup [118], with per-base alignment quality disabled (−B). A sync file was created using popoolation mpileup2sync (parameters: --min-qual 20), which contained the nucleotide composition of each sex at each position in the reference genome. Using this sync file, F_{ST}, SNPs, and coverage between the two sexes were calculated for all reference positions in a 50 kb sliding window, with an output point every 1,000 bp to identify sex-specific SNP-enriched regions.

4.4 RNA-seq

For RNA-seq experiments, 60 individuals from one full-sib family of *M. macrocephalus* were used. The fish were produced and maintained as described in section **4.2 Linkage mapping**. At 150 days post-fertilization, when the period of sex differentiation had recently occurred according to previous experiments in this species *(unpublished data*), the two gonads and kidneys of each fish were immediately dissected. Fish were euthanized by an overdose of benzocaine anesthetic (2%) for sampling. One gonad was stored in RNAlater (Thermo Fischer Scientific) for RNA extraction, and the other was fixed for 24 hours in Karnovsky's solution [119] and then stored in 70% ethanol for phenotypic sex identification via microscopy. The sex of each fish was verified by cytogenetic analysis, as detailed in section **4.2 Linkage mapping.** Phenotypic sex was determined through gonadal histology as described by [120].

After phenotypic and genotypic sex identification, the samples were clustered into two pools: ZZ males and ZW females. Each pool had three biological replicates, each consisting of 10 gonads, resulting in 6 libraries for RNA sequencing. RNA was extracted from each pool using the RNeasy Micro Kit (Qiagen). The integrity (RIN > 7) and concentration (ng/µl) were accessed using the Bioanalyzer 2100 (Agilent). Library construction and sequencing were then performed by BGI Genomics (Shenzhen, China) using the BGISEQ-500 platform 100 bp paired-end reads.

Raw read quality was accessed using FastQC. Adapters and poor-quality reads were trimmed using Trimmomatic [97] with the parameters LEADING:20 TRAILING:20 SLIDINGWINDOW:4:20 MINLEN:36). Trimmed reads were pseudo-aligned against mRNA sequences obtained from the *M. macrocephalus* genome (GCA_021613375.1) using kallisto [121]. A matrix with estimated counts of transcripts abundance was exported using R/tximport [122]. Differential expression analysis was performed using R/DESeq2 [123], with the design

formula ~ sex. Transcripts with False Discovery Rate (FDR) adjusted p-values ≤ 0.01 were considered differentially expressed. Transcripts with Log Fold Change (LFC) ≥ 1 were considered up-regulated in males and transcripts with LFC ≤ -1 were considered up-regulated in females.

Abbreviations

BMPs: Bone morphogenetic proteins; BUSCO: Benchmarking Universal Single Copy Orthologs; CLR: continuous long reads; ddRADseq: Double Digest Restriction Site Associated DNA Sequencing; DE: differentially expressed; FISH: Fluorescence in situ hybridization; F_{st}: fixation index; GO: gene ontology; HMW: high molecular weight; JBAT: Juicebox Assembly Tools; LG: Linkage Group; LINE: long interspersed nuclear elements; LOD: Logarithm of Odds; LTR: long terminal repeats; MSD: master sex determining; NCBI: National Center for Biotechnology Information; PacBio: Pacific Biosciences; PAR: Pseudoautosomal region; PCA: Principal component analysis; QV: Consensus Quality Value; sat DNA: Satellite DNAs; SINE: short interspersed nuclear elements; SMRT: single molecule real time; T2T: Telomere to Telomere consortium; TE: transposable elements.

Additional Files

supplementary_material_tables.docx – Supplementary tables.

supplementary_material_figures.pdf – Supplementary figures.

supplementary_material_gene_description.xlsx – Table containing gene ID, chromosome, position (bp), gene symbol and description.

supplementary_material_DE_results.xlsx – Table containing Differential Expression analysis results (gene ID, log_2 fold change, *p*adj, chromosome, position (bp), gene symbol and description).

Declarations

Ethics approval

This study was conducted in strict accordance with the recommendations of the National Council for Control of Animal Experimentation (CONCEA) (Brazilian Ministry of Science, Technology, and Innovation) and was approved by the Ethics Committee on Animal Use (CEUA number 4936/20) of Faculdade de Ciências Agrárias e Veterinárias, UNESP, Campus Jaboticabal, SP, Brazil.

Data Availability

This Whole Genome Shotgun project has been deposited at DDBJ/ENA/GenBank under the accession JAJQXZ000000000. The version described in this paper is version JAJQXZ010000000. The assembled genome is available at the NCBI with the accession number GCA_021613375.1. All additional supporting data are available in the *GigaScience* repository, GigaDB [124].

Competing interests

The authors declare that they have no competing interests.

Funding

This study was partially financed by the Coordenação de Aperfeiçoamento de Pessoal de Nível Superior (CAPES), under Award Number 88887.467255/2019-00; Conselho Nacional de Desenvolvimento Científico e Tecnológico (CNPq), under Award Number 404386/2021, and the Brazilian Government.

Authors' contributions

DTH, RU and CHSB conceived and designed the study. DTH, RU, RH, YG, FPF, FF, AB and CP supervised the research. CHSB wrote the manuscript with inputs from DTH. CHSB, MUS, AV and DTH performed bioinformatic analysis. DTH, RU, FPF, FF, RH and YG provided funding. CHSB constructed the ddRADseq libraries. CHSB and SM extracted DNA of pool-sex samples. CHSB, DTH, JFGA, LVGL, MVF, RBA performed induced spawning of breeders for the ddRADseq experiment. CHSB, DTH, RU and RBA performed cytogenetics analysis. CHSB, AJB and LVGL performed histologic analysis and RNA extraction of RNAseq samples. RSH, AJB, CHSB and LVGL analyzed the histology samples. CHSB, DTH, RU, JFGA, MVF, VAMF, RBA collected data and samples. All authors read and approved the final manuscript.

Acknowledgments

This research article was possible thanks to the scholarship granted from the Brazilian Federal Agency for Support and Evaluation of Graduate Education (CAPES), in the scope of the Program CAPES-PrInt, process number 88887.467255/2019-00. Also, we would like to thank Valdecir Fernandes de Lima and Marcio Roberto Reche for their support in collecting the samples and handling the fish.

References

1. Fricke R, Eschmeyer WN, Fong JD: SPECIES BY FAMILY/SUBFAMILY. http://researcharchive.calacademy.org/research/ichthyology/catalog/SpeciesByFamily.asp (2024). Accessed 2024 Jun 19.

2. Garavello JC, Britski HA. Check List of the Freshwater Fishes of South and Central America: Family Anostomidae. Reis RE, Kullander SO, Ferraris Júnior CJ, editors. Porto Alegre: EDIPUCRS; 2003.

3. Reynalte-Tataje D, Esquivel B, Esquivel J, Zaniboni-Filho E. Induced reproduction of piauçu, Leporinus macrocephalus Garavello and Britski, 1988 (Characiformes, Anostomidae). *BOLETIM DO INSTITUTO DE PESCA*. 282002;

4. Ramirez JL, Birindelli JLO, Galetti PM. A new genus of Anostomidae (Ostariophysi: Characiformes): Diversity, phylogeny and biogeography based on cytogenetic, molecular and morphological data. *Mol Phylogenet Evol*. Elsevier Inc.; 2017; doi: 10.1016/j.ympev.2016.11.012.

5. Wright AE, Dean R, Zimmer F, Mank JE. How to make a sex chromosome. *Nat Commun*. 2016; doi: 10.1038/ncomms12087.

6. Galetti PM, Cesar ACG, Venere PC. Heterochromatin and NORs variability in Leporinus fish (Anostomidae, Characiformes). *Caryologia*. 1991; doi: 10.1080/00087114.1991.10797193.

7. Galetti, Jr. PM, Foresti F, Bertqllo LAC, Filho M. Heteromorphic sex chromosomes in three species of the genus Leporinus (Pisces, Anostomidae). *Cytogenet Genome Res*. 1981; doi: 10.1159/000131562.

8. Galetti PM, Lima NRW, Venere PC. A Monophyletic ZW Sex Chromosome System in Leporinus (Anostomidae, Characiformes). *Cytologia (Tokyo)*. 1995; doi: 10.1508/CYTOLOGIA.60.375.

9. Venere PC, Ferreira IA, Martins C, Galetti PM. A novel ZZ/ZW sex chromosome system for the genus Leporinus (Pisces, Anostomidae, Characiformes). *Genetica*. 2004; doi: 10.1023/B:GENE.0000019936.40970.7A/METRICS.

10. Navarro RD, Pinto da Matta SL, Teixeira Lanna EA, Lopes Donzele J, Souza Rodrigues S, Fortes da Silva R, et al.. Níveis de energia digestível na dieta de piauçu (Leporinus macrocephalus) no desenvolvimento testicular em estágio pós-larval. *Zootec Trop*. 2006. 24:153–632006;

11. Garavello JC, Britski HA. Leporinus macrocephalus sp. da bacia do rio Paraguai (Ostariophysi, Anostomidae). *Naturalia*. 1988. 13:67–741988;

12. Utsunomia R, Silva DMZ de A, Ruiz-Ruano FJ, Goes CAG, Melo S, Ramos LP, et al.. Satellitome landscape analysis of Megaleporinus macrocephalus (Teleostei, Anostomidae) reveals intense accumulation of satellite sequences on the heteromorphic sex chromosome. *Sci Rep*. 2019; doi: 10.1038/s41598-019-42383-8.

13. Hashimoto DT, Mendonça FF, Senhorini JA, Bortolozzi J, de Oliveira C, Foresti F, et al.. Identification of hybrids between Neotropical fish Leporinus macrocephalus and Leporinus elongatus by PCR-RFLP and multiplex-PCR: Tools for genetic monitoring in aquaculture. *Aquaculture*. 2010; doi: 10.1016/j.aquaculture.2009.11.015.

14. De Carvalho DC, Oliveira DAA, Pompeu PS, Leal CG, Oliveira C, Hanner R. Deep barcode divergence in Brazilian freshwater fishes: The case of the São Francisco River basin. *Mitochondrial DNA*. 2011; doi: 10.3109/19401736.2011.588214.

15. Ramirez JL, Birindelli JL, Carvalho DC, Affonso PRAM, Venere PC, Ortega H, et al.. Revealing hidden diversity of the underestimated neotropical ichthyofauna: DNA barcoding in the recently described genus Megaleporinus (characiformes: Anostomidae). *Front Genet*. 2017; doi: 10.3389/fgene.2017.00149.

16. Almeida MS, Moraes PSS, Nascimento MHS, Birindelli JLO, Assega FM, Barros MC, et al.. New records of the occurrence of Megaleporinus macrocephalus (Garavello & Britski, 1988) (Characiformes, Anostomidae) from the basins of the Itapecuru and Mearim rivers in Maranhão, Northeastern Brazil. *Brazilian Journal of Biology*. 2022; doi: 10.1590/1519-6984.232868.

17. Ferreira IA, Oliveira C, Venere PC, Galetti PM, Martins C. 5S rDNA variation and its phylogenetic inference in the genus Leporinus (Characiformes: Anostomidae). *Genetica*. 2007; doi: 10.1007/s10709-006-0005-6.

18. Avelino GS, Britski HA, Foresti F, Oliveira C. Molecular identification of Leporinus from the south portion of South America. *DNA Barcodes*. 2016; doi: 10.1515/dna-2015-0013.

19. Morelli KA, Revaldaves E, Oliveira C, Foresti F. Isolation and characterization of eight microsatellite loci in Leporinus macrocephalus (Characiformes: Anostomidae) and cross-species amplification. *Mol Ecol Notes*. 2007; doi: 10.1111/j.1471-8286.2006.01484.x.

20. De Silva DMZA, Utsunomia R, Ruiz-Ruano FJ, Daniel SN, Porto-Foresti F, Hashimoto DT, et al.. High-throughput analysis unveils a highly shared satellite DNA library among three species of fish genus Astyanax. *Sci Rep*. 2017; doi: 10.1038/s41598-017-12939-7.

21. Serrano-Freitas ÉA, Silva DMZA, Ruiz-Ruano FJ, Utsunomia R, Araya-Jaime C, Oliveira C, et al.. Satellite DNA content of B chromosomes in the characid fish Characidium gomesi supports their origin from sex chromosomes. *Molecular Genetics and Genomics*. 2020; doi: 10.1007/s00438-019-01615-2.

22. Crepaldi C, Martí E, Gonçalves ÉM, Martí DA, Parise-Maltempi PP. Genomic Differences Between the Sexes in a Fish Species Seen Through Satellite DNAs. *Front Genet*. 2021; doi: 10.3389/fgene.2021.728670.

23. Charlesworth D, Charlesworth B, Marais G. Steps in the evolution of heteromorphic sex chromosomes. *Heredity*. 2005; doi: 10.1038/sj.hdy.6800697.

24. Pollard MO, Gurdasani D, Mentzer AJ, Porter T, Sandhu MS. Long reads: their purpose and place. *Hum Mol Genet*. Oxford Academic; 2018; doi: 10.1093/HMG/DDY177.

25. Ramos L, Antunes A. Decoding sex: Elucidating sex determination and how high-quality genome assemblies are untangling the evolutionary dynamics of sex chromosomes. *Genomics*. 2022; doi: 10.1016/J.YGENO.2022.110277.

26. Rhie A, McCarthy SA, Fedrigo O, Damas J, Formenti G, Koren S, et al.. Towards complete and error-free genome assemblies of all vertebrate species. *Nature 2021 592:7856*. 2021; doi: 10.1038/s41586-021-03451-0.

27. Tomaszkiewicz M, Medvedev P, Makova KD. Y and W Chromosome Assemblies: Approaches and Discoveries. *Trends in Genetics*. 2017; doi: 10.1016/j.tig.2017.01.008.

28. Xue L, Gao Y, Wu M, Tian T, Fan H, Huang Y, et al.. Telomere-to-telomere assembly of a fish Y chromosome reveals the origin of a young sex chromosome pair. *Genome Biology* 2021; doi: 10.1186/S13059-021-02430-Y.

29. Peichel CL, McCann SR, Ross JA, Naftaly AFS, Urton JR, Cech JN, et al.. Assembly of the threespine stickleback Y chromosome reveals convergent signatures of sex chromosome evolution. *Genome Biology*. 2020; doi: 10.1186/S13059-020-02097-X.

30. Rafati N, Chen J, Herpin A, Pettersson ME, Han F, Feng C, et al.. Reconstruction of the birth of a male sex chromosome present in Atlantic herring. *Proc Natl Acad Sci*. 2020; doi: 10.1073/PNAS.2009925117/-/DCSUPPLEMENTAL.

31. Li M, Zhang R, Fan G, Xu W, Zhou Q, Wang L, et al.. Reconstruction of the Origin of a Neo-Y Sex Chromosome and Its Evolution in the Spotted Knifejaw, Oplegnathus punctatus. *Mol Biol Evol*. 2021; doi: 10.1093/MOLBEV/MSAB056.

32. Chen S, Zhang G, Shao C, Huang Q, Liu G, Zhang P, et al.. Whole-genome sequence of a flatfish provides insights into ZW sex chromosome evolution and adaptation to a benthic lifestyle. *Nat Genet*. 2014; doi: 10.1038/ng.2890.

33. Xu X wen, Sun P, Gao C, Zheng W, Chen S. Assembly of the poorly differentiated Verasper variegatus W chromosome by different sequencing technologies. *Sci Data*. 2023; doi: 10.1038/s41597-023-02790-z.

34. Chin CS, Peluso P, Sedlazeck FJ, Nattestad M, Concepcion GT, Clum A, et al.. Phased diploid genome assembly with single-molecule real-time sequencing. *Nat Methods*. 2016; doi: 10.1038/nmeth.4035.

35. Porto-Foresti F, Hashimoto DT, Alves AL, Almeida RBC, Senhorini JA, Bortolozzi J, et al.. Cytogenetic markers as diagnoses in the identification of the hybrid between Piau&u (Leporinus macrocephalus) and Piapara (Leporinus elongatus). *Genet Mol Biol*. 2008; doi: 10.1590/s1415-47572008000200005.

36. Rhie A, Walenz BP, Koren S, Phillippy AM. Merqury: Reference-free quality, completeness, and phasing assessment for genome assemblies. *Genome Biol*. 2020; doi: 10.1186/s13059-020-02134-9.

37. Logsdon GA, Vollger MR, Eichler EE. Long-read human genome sequencing and its applications. *Nature Reviews Genetics 2020 21:10*. 2020; doi: 10.1038/s41576-020-0236-x.

38. Hoff KJ, Lange S, Lomsadze A, Borodovsky M, Stanke M. BRAKER1: Unsupervised RNA-Seq-Based Genome Annotation with GeneMark-ET and AUGUSTUS. *Bioinformatics*. 2016; doi: 10.1093/bioinformatics/btv661.

39. Brůna T, Hoff KJ, Lomsadze A, Stanke M, Borodovsky M. BRAKER2: automatic eukaryotic genome annotation with GeneMark-EP+ and AUGUSTUS supported by a protein database. *NAR Genom Bioinform*. 2021; doi: 10.1093/NARGAB/LQAA108.

40. Gabriel L, Hoff KJ, Brůna T, Borodovsky M, Stanke M. TSEBRA: transcript selector for BRAKER. *BMC Bioinformatics*. 2021; doi: 10.1186/S12859-021-04482-0/FIGURES/3.

41. Bateman A, Martin MJ, Orchard S, Magrane M, Agivetova R, Ahmad S, et al.. UniProt: the universal protein knowledgebase in 2021. *Nucleic Acids Res*. 2021; doi: 10.1093/NAR/GKAA1100.

42. Huerta-Cepas J, Szklarczyk D, Heller D, Hernández-Plaza A, Forslund SK, Cook H, et al.. eggNOG 5.0: a hierarchical, functionally and phylogenetically annotated orthology resource based on 5090 organisms and 2502 viruses. *Nucleic Acids Res*. 2019; doi: 10.1093/NAR/GKY1085.

43. Purcell S, Neale B, Todd-Brown K, Thomas L, Ferreira MAR, Bender D, et al.. PLINK: A tool set for whole-genome association and population-based linkage analyses. *Am J Hum Genet*. 2007; doi: 10.1086/519795.

44. Catchen J, Amores A, Bassham S. Chromonomer: A tool set for repairing and enhancing assembled genomes through integration of genetic maps and conserved synteny. *G3: Genes, Genomes, Genetics*. 2020; doi: 10.1534/g3.120.401485.

45. Melo BF, Sidlauskas BL, Near TJ, Roxo FF, Ghezelayagh A, Ochoa LE, et al.. Accelerated Diversification Explains the Exceptional Species Richness of Tropical Characoid Fishes. *Syst Biol*. 2021; doi: 10.1093/SYSBIO/SYAB040.

46. Hilsdorf AWS, Uliano-Silva M, Coutinho LL, Montenegro H, Almeida-Val VMF, Pinhal D. Genome assembly and annotation of the tambaqui (Colossoma macropomum): an emblematic fish of the Amazon River basin. *GigaByte*. 2021. doi: 10.46471/gigabyte.29.

47. Warren WC, Boggs TE, Borowsky R, Carlson BM, Ferrufino E, Gross JB, et al.. A chromosome-level genome of Astyanax mexicanus surface fish for comparing population-specific genetic differences contributing to trait evolution. *Nature Communications*. 2021; doi: 10.1038/s41467-021-21733-z.

48. Gao B, Shen D, Xue S, Chen C, Cui H, Song C. The contribution of transposable elements to size variations between four teleost genomes. *Mob DNA*. 2016; doi: 10.1186/s13100-016-0059-7.

49. Schartl M, Kneitz S, Volkoff H, Adolfi M, Schmidt C, Fischer P, et al.. The Piranha Genome Provides Molecular Insight Associated to Its Unique Feeding Behavior. *Genome Biol Evol*. 2019; doi: 10.1093/GBE/EVZ139.

50. Chalopin D, Naville M, Plard F, Galiana D, Volff JN. Comparative analysis of transposable elements highlights mobilome diversity and evolution in vertebrates. *Genome Biol Evol*. 2015; doi: 10.1093/GBE/EVV005.

51. Warren WC, Boggs TE, Borowsky R, Carlson BM, Ferrufino E, Gross JB, et al.. A chromosome-level genome of Astyanax mexicanus surface fish for comparing population-specific genetic differences contributing to trait evolution. *Nature Communications*. 2021; doi: 10.1038/s41467-021-21733-z.

52. Fowler RF, Bonnewell V, Spann MS, Skinner DM. Sequences of three closely related variants of a complex satellite DNA diverge at specific domains. *Journal of Biological Chemistry*. 1985; doi: 10.1016/s0021-9258(17)39443-7.

53. Plohl M, Meštrović N, Mravinac B. Satellite DNA Evolution. *Genome Dyn*. 2012; doi: 10.1159/000337122.

54. Cechova M. Probably correct: Rescuing repeats with short and long reads. *Genes (Basel)*. 2021; doi: 10.3390/genes12010048.

55. Salzberg SL, Yorke JA. Beware of mis-assembled genomes. *Bioinformatics*. 2005; doi: 10.1093/BIOINFORMATICS/BTI769.

56. Harris RS, Cechova M, Makova KD. Noise-cancelling repeat finder: uncovering tandem repeats in error-prone long-read sequencing data. *Bioinformatics*. 2019; doi: 10.1093/bioinformatics/btz484.

57. Peona V, Kutschera VE, Blom MPK, Irestedt M, Suh A. Satellite DNA evolution in Corvoidea inferred from short and long reads. *Mol Ecol*. 2022; doi: 10.1111/MEC.16484.

58. Zheng Y, Ay F, Keles S. Generative modeling of multi-mapping reads with mhi-c advances analysis of hi-c studies. *Elife*. 2019; doi: 10.7554/ELIFE.38070.

59. Mitsuhashi S, Frith MC, Mizuguchi T, Miyatake S, Toyota T, Adachi H, et al.. Tandem-genotypes: robust detection of tandem repeat expansions from long DNA reads. *Genome Biol*. 2019; doi: 10.1186/S13059-019-1667-6/TABLES/2.

60. Ummat A, Bashir A. Resolving complex tandem repeats with long reads. *Bioinformatics.* 2014; doi: 10.1093/bioinformatics/btu437.

61. Mikheenko A, Bzikadze A V., Gurevich A, Miga KH, Pevzner PA. TandemTools: mapping long reads and assessing/improving assembly quality in extra-long tandem repeats. *Bioinformatics*. 2020; doi: 10.1093/BIOINFORMATICS/BTAA440.

62. Jain C, Rhie A, Hansen NF, Koren S, Phillippy AM. Long-read mapping to repetitive reference sequences using Winnowmap2. *Nat Methods*. 2022. doi: 10.1038/s41592-022-01457-8.

63. Cechova M, Vegesna R, Tomaszkiewicz M, Harris RS, Chen D, Rangavittal S, et al.. Dynamic evolution of great ape Y chromosomes. *PNAS*. 2001; doi: 10.1073/pnas.2001749117/-/DCSupplemental.

64. Rastas P, Paulin L, Hanski I, Lehtonen R, Auvinen P, Brudno M. Lep-MAP: Fast and accurate linkage map construction for large SNP datasets. *Bioinformatics*. 2013; doi: 10.1093/bioinformatics/btt563.

65. Varela ES, Bekaert M, Ganeco-Kirschnik LN, Torati LS, Shiotsuki L, de Almeida FL, et al.. A high-density linkage map and sex-linked markers for the Amazon Tambaqui Colossoma macropomum. *BMC Genomics*. 2021; doi: 10.1186/S12864-021-08037-8/TABLES/4.

66. Ariede RB, Freitas M V., Lira LVG, Agudelo JFG, Borges CHS, Mastrochirico-Filho VA, et al.. Linkage map for chromosome-level genome anchoring and genome-wide association study for resistance to Aeromonas hydrophila in Colossoma macropomum. *Aquaculture*. 2022; doi: 10.1016/J.AQUACULTURE.2022.738462.

67. Lien S, Gidskehaug L, Moen T, Hayes BJ, Berg PR, Davidson WS, et al.. A dense SNP-based linkage map for Atlantic salmon (Salmo salar) reveals extended chromosome homeologies and striking differences in sex-specific recombination patterns. *BMC Genomics*. 2011; doi: 10.1186/1471-2164-12-615.

68. Sakamoto T, Danzmann RG, Gharbi K, Howard P, Ozaki A, Kean Khoo S, et al.. A Microsatellite Linkage Map of Rainbow Trout (Oncorhynchus mykiss) Characterized by Large Sex-Specific Differences in Recombination Rates. *Genetics*. 2000. doi: 10.1093/genetics/155.3.1331.

69. Castaño-Sánchez C, Fuji K, Ozaki A, Hasegawa O, Sakamoto T, Morishima K, et al.. A second generation genetic linkage map of Japanese flounder (Paralichthys olivaceus). *BMC Genomics*. 2010; doi: 10.1186/1471-2164-11-554.

70. Reid DP, Smith CA, Rommens M, Blanchard B, Martin-Robichaud D, Reith M. A genetic linkage map of Atlantic halibut (Hippoglossus hippoglossus L.). *Genetics*. 2007; doi: 10.1534/genetics.107.075374.

71. Song W, Li Y, Zhao Y, Liu Y, Niu Y, Pang R, et al.. Construction of a High-Density Microsatellite Genetic Linkage Map and Mapping of Sexual and Growth-Related Traits in Half-Smooth Tongue Sole (Cynoglossus semilaevis). *PLoS One*. 2012; doi: 10.1371/journal.pone.0052097.

72. Bouza C, Hermida M, Pardo BG, Fernández C, Fortes GG, Castro J, et al.. A microsatellite genetic map of the turbot (Scophthalmus maximus). *Genetics*. 2007; doi: 10.1534/genetics.107.075416.

73. Guerrero-Cózar I, Perez-Garcia C, Benzekri H, Sánchez JJ, Seoane P, Cruz F, et al.. Development of whole-genome multiplex assays and construction of an integrated genetic map using SSR markers in Senegalese sole. *Sci Rep*. 2020; doi: 10.1038/s41598-020-78397-w.

74. de los Ríos-Pérez L, Nguinkal JA, Verleih M, Rebl A, Brunner RM, Klosa J, et al.. An ultra-high density SNP-based linkage map for enhancing the pikeperch (Sander lucioperca) genome assembly to chromosome-scale. *Sci Rep*. 2020; doi: 10.1038/s41598-020-79358-z.

75. Smith SR, Normandeau E, Djambazian H, Nawarathna PM, Berube P, Muir AM, et al.. A chromosome-anchored genome assembly for Lake Trout (Salvelinus namaycush). *Mol Ecol Resour*. 2022; doi: 10.1111/1755-0998.13483.

76. Carey SB, Lovell JT, Jenkins J, Leebens-Mack J, Schmutz J, Wilson MA, et al.. Representing sex chromosomes in genome assemblies. *Cell Genomics*. 2022; doi: 10.1016/J.XGEN.2022.100132.

77. Liu J, Wang Z, Li J, Xu L, Liu J, Feng S, et al.. A new emu genome illuminates the evolution of genome configuration and nuclear architecture of avian chromosomes. *Genome Res*. 2021; doi: 10.1101/gr.271569.120.

78. Skaletsky H, Kuroda-Kawaguchi T, Minx PJ, Cordum HS, Hillier L, Brown LG, et al.. The male-specific region of the human Y chromosome is a mosaic of discrete sequence classes. *Nature.* 2003. doi: 10.1038/nature01722

79. Schneider VA, Graves-Lindsay T, Howe K, Bouk N, Chen HC, Kitts PA, et al.. Evaluation of GRCh38 and de novo haploid genome assemblies demonstrates the enduring quality of the reference assembly. *Genome Res*. 2017; doi: 10.1101/gr.213611.116.

80. Rhie A, Nurk S, Cechova M, Hoyt SJ, Taylor DJ, Altemose N, et al.. The complete sequence of a human Y chromosome. *Nature*. 2023; doi: 10.1038/s41586-023-06457-y.

81. Nurk S, Koren S, Rhie A, Rautiainen M, Bzikadze A V, Mikheenko A, et al.. The complete sequence of a human genome. *Science*. 2022; doi: https://doi.org/10.1126/science.abj6987.

82. Miga KH, Koren S, Rhie A, Vollger MR, Gershman A, Bzikadze A, et al.. Telomere-to-telomere assembly of a complete human X chromosome. *Nature*. 2020; doi: 10.1038/s41586-020-2547-7.

83. Rautiainen M, Nurk S, Walenz BP, Logsdon GA, Porubsky D, Rhie A, et al.. Telomere-to-telomere assembly of diploid chromosomes with Verkko. *Nature Biotechnology* |. 2023; doi: 10.1038/s41587-023-01662-6.

84. Makova KD, Pickett BD, Harris RS, Hartley GA, Cechova M, Pal K, et al.. The complete sequence and comparative analysis of ape sex chromosomes. *Nature*. 2024; doi: 10.1038/s41586-024-07473-2.

85. Hu Y, Wang B, Du H. A review on sox genes in fish. *Reviews in Aquaculture*. 2021; doi: 10.1111/raq.12554.

86. Porgy B, Schlegeli A, Wu G-C, Chang C-F. wnt4 Is Associated with the Development of Ovarian Tissue in the Protandrous Black Porgy, Acanthopagrus schlegeli. *Biol Reprod*. 2009; doi: 10.1095/BIOLREPROD.109.077362.

87. Pan Q, Kay T, Depincé A, Adolfi M, Schartl M, Guiguen Y, et al.. Evolution of master sex determiners: TGF-β signalling pathways at regulatory crossroads. *Philosophical Transactions of the Royal Society B*. 2021; doi: 10.1098/RSTB.2020.0091.

88. Pangas SA. Bone morphogenetic protein signaling transcription factor (SMAD) function in granulosa cells. *Mol Cell Endocrinol*. 2012; doi: 10.1016/J.MCE.2011.06.021.

89. Ross A, Munger S, Capel B. Bmp7 Regulates Germ Cell Proliferation in Mouse Fetal Gonads. *Sexual Development*. 2007; doi: 10.1159/000100034.

90. Ogino Y, Hirakawa I, Inohaya K, Sumiya E, Miyagawa S, Denslow N, et al.. Bmp7 and Lef1 Are the Downstream Effectors of Androgen Signaling in Androgen-Induced Sex Characteristics Development in Medaka. *Endocrinology*. 2014; doi: 10.1210/en.2013-1507.

91. Kamiya T, Kai W, Tasumi S, Oka A, Matsunaga T, Mizuno N, et al.. A trans-species missense SNP in Amhr2 is associated with sex determination in the tiger Pufferfish, Takifugu rubripes (Fugu). *PLoS Genet*. 2012; doi: 10.1371/journal.pgen.1002798.

92. Feron R, Zahm M, Cabau C, Klopp C, Roques C, Bouchez O, et al.. Characterization of a Y-specific duplication/insertion of the anti-Mullerian hormone type II receptor gene based on a chromosome-scale genome assembly of yellow perch, Perca flavescens. *Mol Ecol Resour*. 2020; doi: 10.1111/1755-0998.13133.

93. Wen M, Pan Q, Jouanno E, Montfort J, Zahm M, Cabau C, et al.. An ancient truncated duplication of the anti-Müllerian hormone receptor type 2 gene is a potential conserved master sex determinant in the Pangasiidae catfish family. *Mol Ecol Resour*. 2022; doi: 10.1111/1755-0998.13620.

94. Terao M, Ogawa Y, Takada S, Kajitani R, Okuno M, Mochimaru Y, et al.. Turnover of mammal sex chromosomes in the Sry-deficient Amami spiny rat is due to male-specific upregulation of Sox9. *Proc Natl Acad Sci*. 2022; doi: 10.1073/pnas.2211574119.

95. Bertollo LAC, Moreira–Filho O, Galetti PM. Cytogenetics and taxonomy: considerations based on chromosome studies of freshwater fish. *J Fish Biol*. 1986; doi: 10.1111/j.1095-8649.1986.tb05153.x.

96. Sumner AT. A simple technique for demonstrating centromeric heterochromatin. *Exp Cell Res*. 1972; doi: 10.1016/0014-4827(72)90558-7.

97. Bolger AM, Lohse M, Usadel B. Trimmomatic: a flexible trimmer for Illumina sequence data. *Bioinformatics*. 2014; doi: 10.1093/BIOINFORMATICS/BTU170.

98. Marçais G, Kingsford C. A fast, lock-free approach for efficient parallel counting of occurrences of k-mers. *Bioinformatics*. 2011; doi: 10.1093/BIOINFORMATICS/BTR011.

99. Vurture GW, Sedlazeck FJ, Nattestad M, Underwood CJ, Fang H, Gurtowski J, et al.. GenomeScope: Fast reference-free genome profiling from short reads. *Bioinformatics*. 2017; doi: 10.1093/bioinformatics/btx153.

100. Kolmogorov M, Yuan J, Lin Y, Pevzner PA. Assembly of long, error-prone reads using repeat graphs. *Nat Biotechnol*. 2019; doi: 10.1038/s41587-019-0072-8.

101. Ruan J, Li H. Fast and accurate long-read assembly with wtdbg2. *Nat Methods*. 2020; doi: 10.1038/s41592-019-0669-3.

102. Koren S, Walenz BP, Berlin K, Miller JR, Bergman NH, Phillippy AM. Canu: Scalable and accurate long-read assembly via adaptive κ-mer weighting and repeat separation. *Genome Res*. 2017; doi: 10.1101/gr.215087.116.

103. Zimin A V., Salzberg SL. The genome polishing tool POLCA makes fast and accurate corrections in genome assemblies. *PLoS Comput Biol*. 2020; doi: 10.1371/journal.pcbi.1007981.

104. Ziminid A V, Salzbergid SL. The SAMBA tool uses long reads to improve the contiguity of genome assemblies. Shao M, editor. *PLoS Comput Biol*. 2022; doi: 10.1371/JOURNAL.PCBI.1009860.

105. Dudchenko O, Batra SS, Omer AD, Nyquist SK, Hoeger M, Durand NC, et al.. De novo assembly of the Aedes aegypti genome using Hi-C yields chromosome-length scaffolds. *Science*. 2017; doi: 10.1126/science.aal3327.

106. Durand NC, Shamim MS, Machol I, Rao SSP, Huntley MH, Lander ES, et al.. Juicer Provides a One-Click System for Analyzing Loop-Resolution Hi-C Experiments. *Cell Syst*. 2016; doi: 10.1016/j.cels.2016.07.002.

107. Manni M, Berkeley MR, Seppey M, Simão FA, Zdobnov EM. BUSCO Update: Novel and Streamlined Workflows along with Broader and Deeper Phylogenetic Coverage for Scoring of Eukaryotic, Prokaryotic, and Viral Genomes. *Mol Biol Evol*. 2021; doi: 10.1093/MOLBEV/MSAB199.

108. Flynn JM, Hubley R, Goubert C, Rosen J, Clark AG, Feschotte C, et al.. RepeatModeler2 for automated genomic discovery of transposable element families. *PNAS*. 2020; doi: 10.1186/s13059-018-1577-z.

109. Kriventseva E V, Kuznetsov D, Tegenfeldt F, Manni M, Dias R, Simão FA, et al.. OrthoDB v10: sampling the diversity of animal, plant, fungal, protist, bacterial and viral genomes for evolutionary and functional annotations of orthologs. *Nucleic Acids Res*. 2018; doi: 10.1093/nar/gky1053.

110. Cantalapiedra CP, Herņandez-Plaza A, Letunic I, Bork P, Huerta-Cepas J. eggNOG-mapper v2: Functional Annotation, Orthology Assignments, and Domain Prediction at the Metagenomic Scale. *Mol Biol Evol*. 2021; doi: 10.1093/MOLBEV/MSAB293.

111. Götz S, García-Gómez JM, Terol J, Williams TD, Nagaraj SH, Nueda MJ, et al.. High-throughput functional annotation and data mining with the Blast2GO suite. *Nucleic Acids Res*. 2008; doi: 10.1093/NAR/GKN176.

112. Manni M, Berkeley MR, Seppey M, Simão FA, Zdobnov EM. BUSCO Update: Novel and Streamlined Workflows along with Broader and Deeper Phylogenetic Coverage for Scoring of Eukaryotic, Prokaryotic, and Viral Genomes. *Mol Biol Evol*. 2021; doi: 10.1093/MOLBEV/MSAB199.

113. Foresti F, Toledo LFA, Toledo SA. Polymorphic nature of nucleolus organizer regions in fishes. *Cytogenet Genome Res*. 1981; doi: 10.1159/000131639.

114. Peterson BK, Weber JN, Kay EH, Fisher HS, Hoekstra HE. Double digest RADseq: An inexpensive method for de novo SNP discovery and genotyping in model and non-model species. *PLoS One*. 2012; doi: 10.1371/journal.pone.0037135.

115. Catchen J, Hohenlohe PA, Bassham S, Amores A, Cresko WA. Stacks: An analysis tool set for population genomics. *Mol Ecol*. 2013; doi: 10.1111/mec.12354.

116. Rastas P. Lep-MAP3: Robust linkage mapping even for low-coverage whole genome sequencing data. *Bioinformatics*. 2017; doi: 10.1093/bioinformatics/btx494.

117. Li H, Durbin R. Fast and accurate long-read alignment with Burrows-Wheeler transform. *Bioinformatics*. Bioinformatics; 2010; doi: 10.1093/bioinformatics/btp698.

118. Danecek P, Bonfield JK, Liddle J, Marshall J, Ohan V, Pollard MO, et al. Twelve years of SAMtools and BCFtools. *GigaScience.* 2021,10(2):giab008. doi: 10.1093/gigascience/giab008.

119. Karnovsky MJ. A formaldehyde-glutaraldehyde fixative of high osmolality for use in electron microscopy. *Journal cell biology*. 27:137–81985;

120. Cornelio D, Castro JP, Santos MH, Vicari MR, de Almeida MC, Moreira-Filho O, et al.. Hermaphroditism can compensate for the sex ratio in the Astyanax scabripinnis species complex (Teleostei: Characidae): expanding the B chromosome study model. *Rev Fish Biol Fish*. 2017; doi: 10.1007/S11160-017-9488-8/FIGURES/3.

121. Bray NL, Pimentel H, Melsted P, Pachter L. Near-optimal probabilistic RNA-seq quantification. *Nature Biotechnology*. 2016; doi: 10.1038/nbt.3519.

122. Soneson C, Love MI, Robinson MD, Floor SN. Differential analyses for RNA-seq: transcript-level estimates improve gene-level. *F1000Res.* 2015. doi: 10.12688/f1000research.7563.2.

123. Love MI, Huber W, Anders S. Moderated estimation of fold change and dispersion for RNA-seq data with DESeq2. *Genome Biol*. 2014; doi: 10.1186/S13059-014-0550-8/FIGURES/9.

124. de Souza Borges CH, Utsunomia R, Varani AM, Uliano-Silva M, Guerra Lira LV, Butzge AJ, et al. Supporting data for "De novo assembly and characterization of a highly degenerated ZW sex chromosome in the fish Megaleporinus macrocephalus" GigaScience Database. 2024. https://doi.org/10.5524/102584.

Figure1

Click here to access/download;Figu

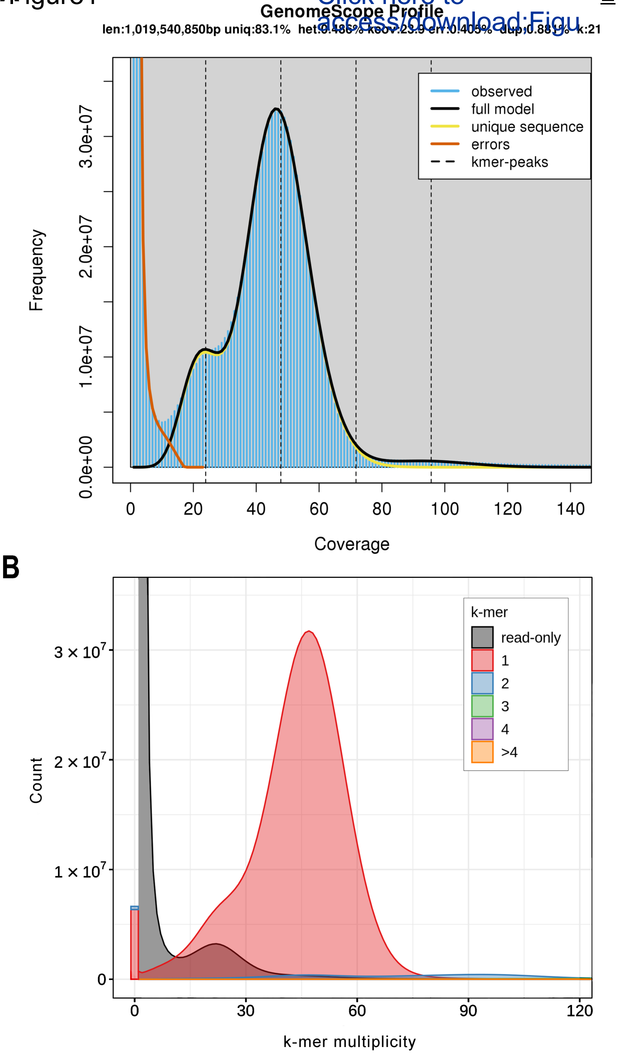

Figure2

Click here to access/downloa

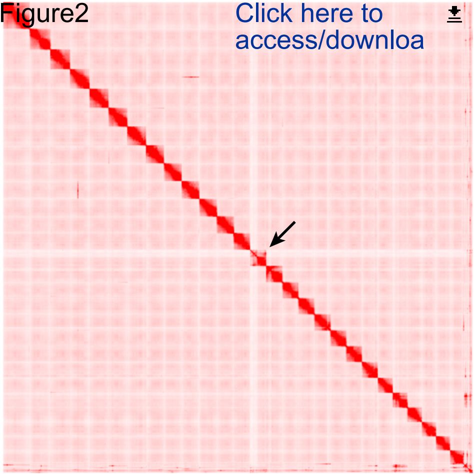

Figure3

Click here to access/download;Figure;Figure3.pdf

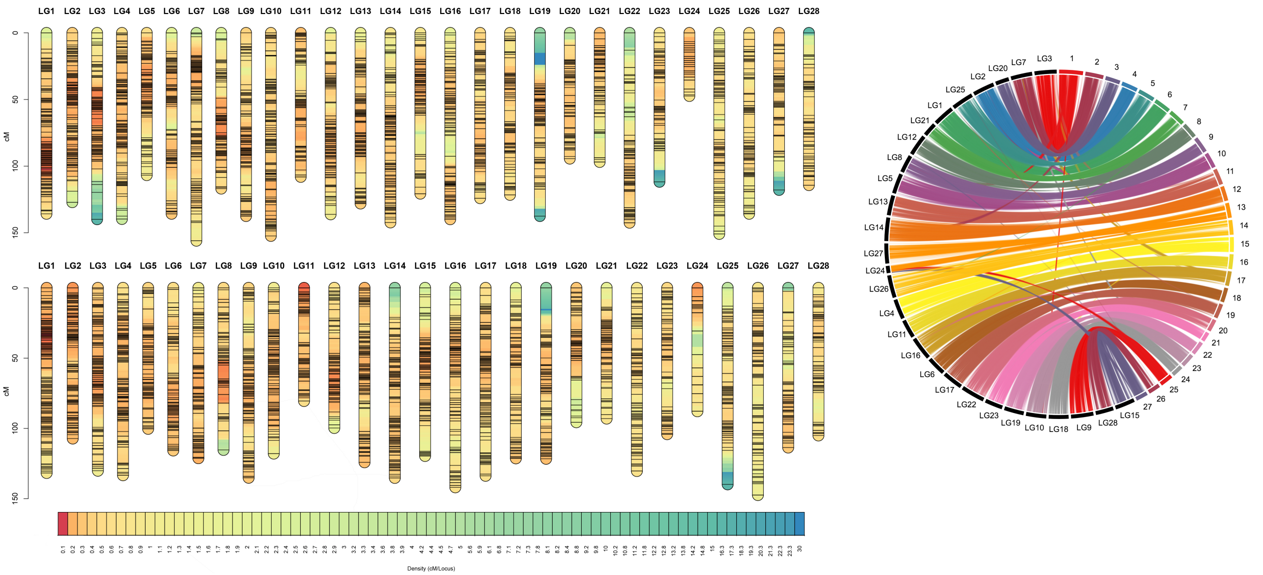

Figure4

Click here to access/download;Fig

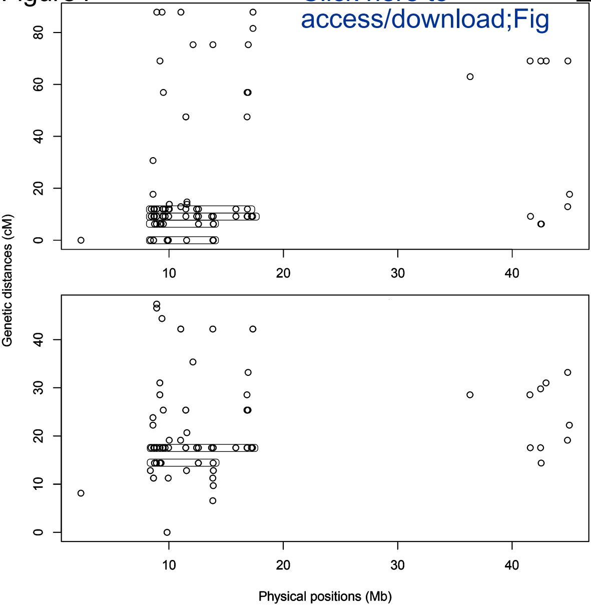

Figure5

Click here to access/download;Figure;Figure5.pdf

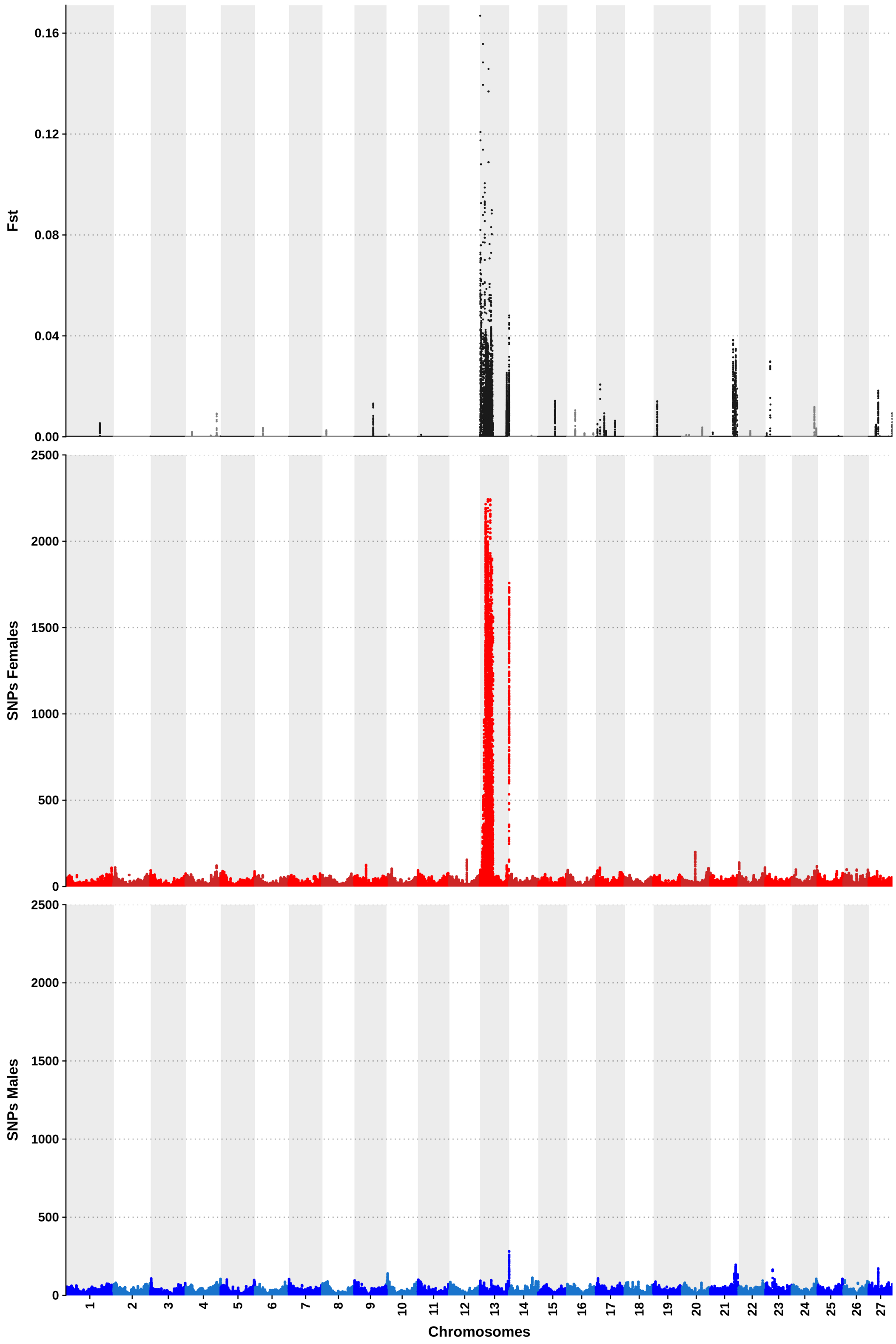

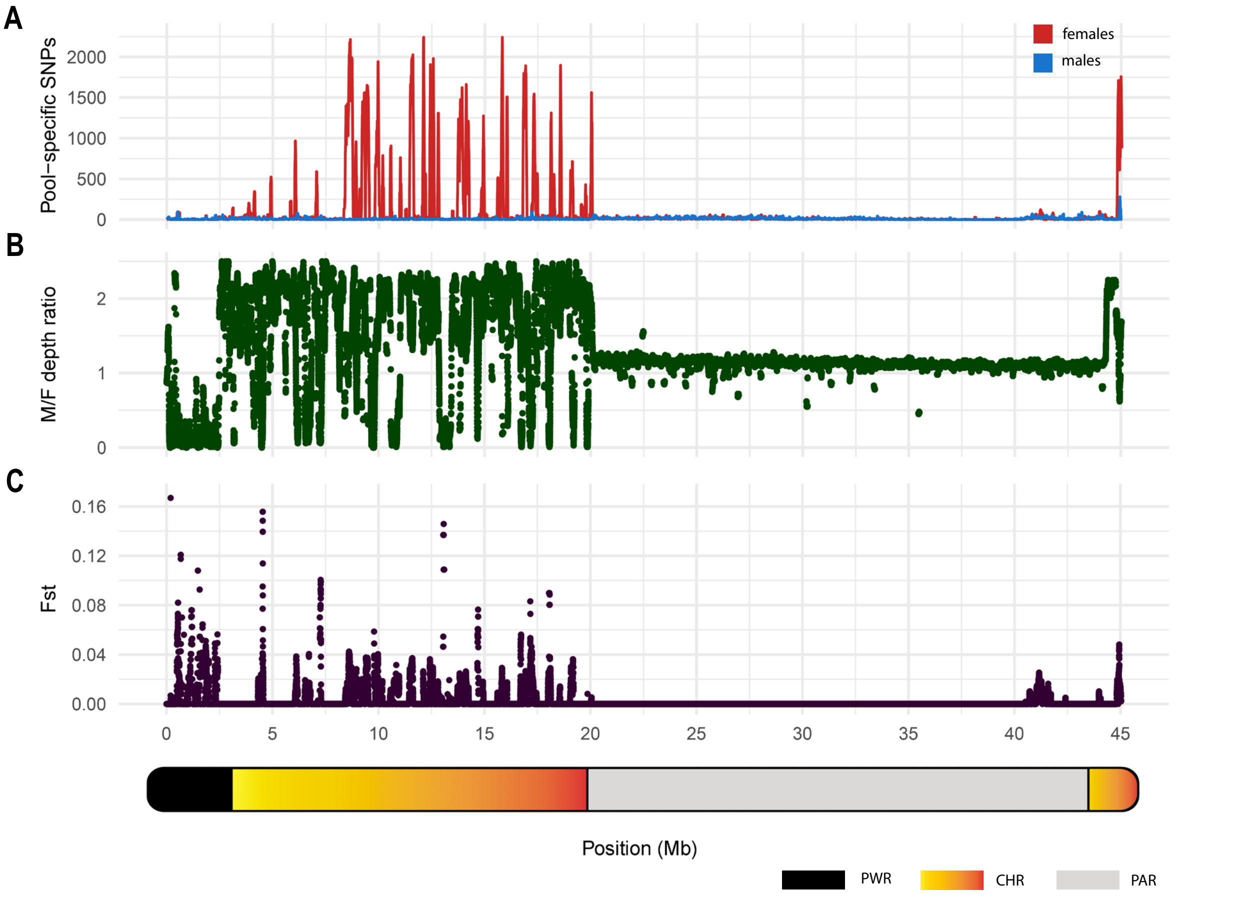
A
females
males
Pool−specific SNPs
2000
1500
1000
500
0
B
M/F depth ratio
2
1
0
C
Fst
0.16
0.12
0.08
0.04
0.00
0
5
10
15
20
25
30
35
40
45
Position (Mb)
PWR
CHR
PAR

Figure7

Click here to access/download;Figur

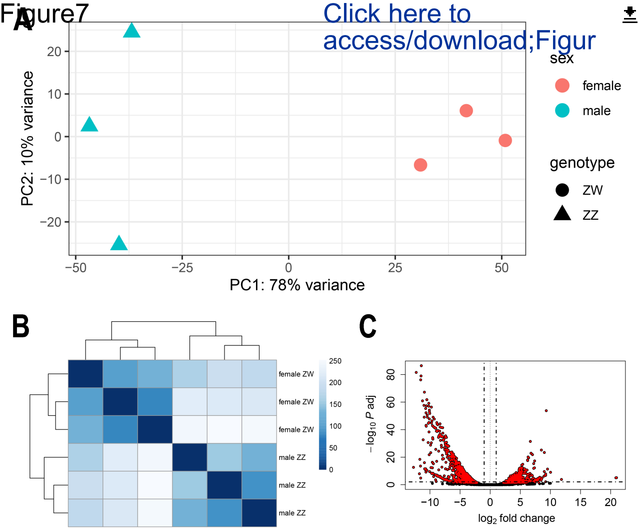

Click here to access/download
Supplementary Material
supplementary_material_tables.docx

Supplementary Material Figures

Click here to access/download
Supplementary Material
supplementary_material_figures.pdf

Supplementary Material Gene Description

Click here to access/download
Supplementary Material
supplementary_material_gene_description.xlsx

Supplementary Material Differential Expression Analysis Results

Click here to access/download
Supplementary Material
supplementary_material_DE_results.xlsx

Personal Cover Click here to access/download;Personal Cover;letter.pdf

UNIVERSIDADE ESTADUAL PAULISTA
"JÚLIO DE MESQUITA FILHO"
Centro de Aquicultura - CAUNESP

September, 07, 2024

Dear Dr. Zhang,

We are resubmitting our revised manuscript titled "De novo assembly and characterization of a highly degenerated ZW sex chromosome in the fish *Megaleporinus macrocephalus*" (GIGA-D-24-00015R2) for reconsideration for publication in GigaScience.

We are grateful for the valuable feedback provided by you and the reviewer. We have carefully considered all the comments and have made modifications to our manuscript to address the points raised.

The revised manuscript conforms to the journal's style guidelines. Below, we provide a detailed point-by-point response to the reviewer's comments. Please find the modifications colored in red in the revised manuscript.

Yours sincerely,

Dr. Diogo Teruo Hashimoto
Centro de Aquicultura, UNESP
14884-900 Jaboticabal, SP, Brazil
Phone: 55 16 3209-7477
E-mail: diogo.hashimoto@unesp.br
