# Supplementary material for: De novo assembly and characterization of a highly degenerated ZW sex chromosome in the fish Megaleporinus macrocephalus
Source: Gigascience. 2024 Nov 26;13:giae085. doi: 10.1093/gigascience/giae085 (PMC11590113; doi:10.1093/gigascience/giae085)
Supplement: giae085_Response_to_Reviewer_Comments_Original_Submission [file giae085_response_to_reviewer_comments_original_submission.pdf]

Author's Response To Reviewer Comments

Reviewer reports:

Reviewer #1: The authors assembled a chromosome-level genomic sequence and identified the sex chromosomes of the fish Megaleporinus macrocephalus. This manuscript is potentially interesting because evolution of sex chromosomes and sex-determining genes are one of the most fundamental and popular topics in the evolutionary biology. However, the conceptual advance and the novelty of this study are quite limited. It is another paper adding now one more species to the list of assembled genomes in this fish family. In addition, there is nothing new about the description of the sex chromosomes such as their degenerative signature. Such studies have already been conducted many times and similar conclusions have been reported. Furthermore, the experimental evidence presented appears rather preliminary and is not sufficient to support the claims and interpretations presented in discussion. I am therefore afraid that I have to say that the manuscript does not provide new insights into evolution of sex chromosomes, and thus will not be of sufficient interest to the readers of Gigascience.

1. Overall, the paper was very difficult to read due to a lack of logic structure and many errors, such as confusing between males and females, between chromosomes and linkage groups, and so on.
Thank you for your valuable feedback. We have reviewed and corrected instances where confusion between males and females (lines 224-225) as well as between chromosomes and linkage groups (lines 200-201, 254-256, 472-477), occurred.
2. The introduction is not logically written. It is unclear what is known and to what extent, and why the genome of this species is being determined.

Thank you for your feedback. We have rewritten the introduction to improve its logical flow (lines 2-62).

3. I did not understand why the authors concluded that Chr13 is the W chromosome and not the Z chromosome. They should assemble the Z and W chromosomes separately and confirm them from different information. It is also unclear how they rule out the possibility that the sequences are chimeric. If they really want to reveal the evolutionary process of sex chromosomes, they should use all the data (Hi-C, linkage analysis, Pool-seq, gene information) to compare the
structure of Z and W in detail, including synteny with closely related species.

We apologize for the confusion in the text. We have made some changes to make the text clearer (lines 127-133, 380-386, 490-518).

4. The analysis on sex chromosome gene candidates is too poor. Basic analyses have not been conducted on whether these genes are W-specific, whether they are in both Z and W, whether they have paralogs or not on autosomes, how much sequence variation there is, when and in which cells they are expressed

Thank you for your valuable feedback. We have included additional information to address the points raised (lines 367-394). As stated in our Results (lines 326-327) and Methods sections (lines 749-754), the transcriptomic data were collected from gonadal tissues during the sex differentiation period. Therefore, all candidate sex genes are expressed under these conditions.

5. All of the discussions are superficial and lacking in logic, and it is unclear what they want to discuss.

We have undertaken a thorough revision of the discussion section to address the issues raised (lines 490-518).

6. The figures legends are poorly explained, and contain incorrect information, so I don't understand the meaning of the data at all.

We have thoroughly reviewed all the figure legends and table titles to ensure they are clearer and more detailed (lines 135-136, 205-208, 234-237, 275-277, 291-298, 335-338).

7. This manuscript contained many grammatical errors leading to many confusing statements, and some sentences that were grammatically correct but awkward meaning. I strongly recommend that the authors seek advice of someone with a good knowledge of English, preferably a native speaker.

We have thoroughly revised the text to correct grammatical errors and we sought assistance from a native English speaker with expertise in scientific writing to further refine the language.

Reviewer #2: The authors reported the M. macrocephalus reference genome with a highly degenerated ZW sex chromosome and analyzed the expression pattern of sex
chromosomes. In a word, this work extends our understanding of the mechanisms of sex chromosome evolution of fish species. The interpretation of the results is sound for the most part, and gives enough proof to verify their results. I just have few concerns as followed.

1.On line 54, please confirm it. In the tongue-sole, the size of Z chromosome (21.91Mb) is larger than the W chromosome (16.45Mb).

We apologize for any confusion caused by the original text. Although in the genome assembly of the Chinese tongue-sole, the size of the Z chromosome is larger than the W chromosome, cytogenetic analyses have shown that in metaphase, the W chromosome is actually larger than the Z chromosome. To address this issue and avoid confusion, we have removed the sentence "which explains its larger size compared to the Z chromosome."

2.On line 88, 89 and 116, the numbers mentioned do not correspond with the results in Figure 1A. Please confirm it.

The reviewer is correct, and we appreciate your feedback. We have revised the text (lines 74-76 and 104).

3.In the section on "Gene Prediction and Annotation", a more comprehensive prediction of gene structure can be achieved by combining three methods: de novo prediction, transcriptome prediction, and homology prediction. The results obtained from these three approaches can be integrated using the EVM software, followed by annotation assessment with BUSCO. The method section is somewhat vague and lacks clear logic. For protein prediction, it is advisable to utilize multiple databases, such as SwissProt, InterPro, and Nr, to corroborate evidence from various sources.

Thank you for your comments. We would like to clarify some important points that were not explicitly mentioned in the "Materials and Methods" section.

Indeed, we used the recommended tools in our approach for gene prediction and annotation. Our methodology involved the combination of three distinct methods: de novo prediction, transcriptome-based prediction, and homology-based prediction. We integrated the results from these approaches using the BRAKER and TSEBRA pipelines, which effectively performs the integration of these different sources of evidence for gene prediction.

We acknowledge that the description in the "Methods" section might have lacked clarity and logical flow. We have taken steps to improve this part of the manuscript, now providing a detailed explanation of each step and the rationale behind using these tools (lines 626-639).

Additionally, for protein prediction, we employed a variety of databases, including Uniprot and OrthoDB vertebrata_ obd10, to corroborate evidence from multiple sources and ensure the accuracy and comprehensiveness of our predictions.

Regarding the differences in BUSCO scores between the genome and annotation assessments, it is important to note that these differences can be attributed to several factors, including the thresholds used to delineate between complete and partial genes. For instance, our BUSCO genome assessment results were as follows: 96.2% complete, 95.1% complete and single-copy, 1.1% complete and duplicated, 0.6% fragmented, and 3.2% missing. In contrast, our BUSCO annotation results showed 94.1% complete, 89%

complete and single-copy, 5.1% duplicated, 2.2% fragmented, and 3.7% missing. These variations can arise due to differences in how complete and partial genes are defined in the BUSCO algorithm. Therefore, this result indicates that we have acquired a high-quality annotation.

In summary, we employed a comprehensive approach for gene prediction and annotation that integrates multiple sources of evidence, resulting in a robust and accurate annotation of the gene models. The detailed steps and tools used in our methodology ensure the reliability and completeness of the predicted gene structures.

4.On line 210, there is an error in the caption of Figure 3. Figure 3B should be a colinearity map of the linkage groups and chromosomes.

Thank you for pointing out this error. We have reviewed and corrected the caption for Figure 3 to accurately reflect the content presented (lines 205-208).

5.The SNP sites identified in females may include those from the Z chromosome, linkage group 23 (LG23) will contain SNP information from both the Z and W chromosomes. This could potentially affect the demarcation of the region of sex conflict.

Thank you for pointing this out. We have included a detailed explanation in the manuscript (lines 306-310).

6.On the sex chromosomes, are there candidate genes related to sex differentiation in regions with a high enrichment of specific SNPs? please provide a detailed explanation.

Thank you for your insightful comment. We have made revisions to the manuscript to include these observations (lines 369-376, 387-394).

7.What is the distribution of genes in the Z and W chromosome-specific regions, and what is the gene loss rate?

We have included a detailed explanation in the manuscript (lines 380-386, 490-518).
