# Supplementary material for: De novo assembly and characterization of a highly degenerated ZW sex chromosome in the fish Megaleporinus macrocephalus
Source: Gigascience. 2024 Nov 26;13:giae085. doi: 10.1093/gigascience/giae085 (PMC11590113; doi:10.1093/gigascience/giae085)
Supplement: giae085_Response_to_Reviewer_Comments_Revision_1 [file giae085_response_to_reviewer_comments_revision_1.pdf]

Author's Response To Reviewer Comments

Reviewer reports:
Reviewer #2: This study assembled the genome of Megaleporinus macrocephalus, identified the sex chromosomes and the boundaries of pseudoautosomal region and Z/W-specific region, and statistically analyzed the distribution of genes that are differentially expressed in male and female gonads in sex chromosome. It provides a theoretical foundation for the study of heteromorphic sex chromosomes in fish. I suggest making few revisions to the article as a whole.

1."These species were formerly classified under the genus Leporinus but were reclassified due to the presence of a unique ZZ/ZW sex chromosome system that emerged at least 12 million years ago" Give examples of species with a ZZ/ZW sex chromosome system and explain whether they have homomorphic or heteromorphic sex chromosomes.
Please find the modifications in lines 9-15.

2."it remains the only well-characterized W chromosome in fish" It is not only. The homomorphic Z/W chromosomes of Verasper variegatus has published.
Thank you for the comment. The modified text is in lines 55-58.

3.Does M. macrocephalus exhibit sexual dimorphism, and what economic value does this study foresee for it?
Please see the answer in lines 17-19 and 65-67 of the Background section.

4."The unique molecular yield of PacBio reads was 56 Gb, and the subread N50 length was 32 kb." The first half of the sentence refers to the number of reads, right? If so, the unit "Gb" is not appropriate to use.
Thank you for your comment; you are correct. We have removed the sentence to improve the clarity of the paragraph.

5.The content from lines 77 to 80 can be moved to the Methods section.
Thank you for your suggestion. We have moved the content to the Methods section, and the modifications can be found in lines 628-630.

6.The content from lines 95 to 102 is too verbose. It could serve as a caption for Figure 1B.
Thank you for your suggestion. The content has been moved to the caption of Figure 1B, and the changes can be found in lines 96-105.

7."as both LG24 and LG27 correspond to different regions of the same sex chromosome." However, as can be seen from Figure 3B, part of LG24 also shows collinearity with some regions of chromosomes 25 and 27. This does not adequately prove that LG24 is part of the sex chromosome; it also includes parts of the autosomes.
Thank you for your comment! We have addressed this issue and added the relevant information in lines 238-240.

8."which is undergoing degeneration." What results could corroborate this conclusion?
Thank you for your observation. We have included the supporting information in lines 324-326.

9.Based on information such as linkage groups and defined regions, attempt to assemble the Z and W chromosomes.
We greatly appreciated this suggestion. However, after careful analysis, we decided not to proceed with reassembling the Z and W chromosomes, as it would result in spurious assemblies. This discussion has been included in the Discussion section (lines 523-557).
