# Supplementary material for: De novo assembly and characterization of a highly degenerated ZW sex chromosome in the fish Megaleporinus macrocephalus
Source: Gigascience. 2024 Nov 26;13:giae085. doi: 10.1093/gigascience/giae085 (PMC11590113; doi:10.1093/gigascience/giae085)
Supplement: giae085_Reviewer_1_Report_Original_Submission — Yusuke Takehana -- 3/28/2024 [file giae085_reviewer_1_report_original_submission.pdf]

**Reviewer Report**

**Title: De novo assembly and characterization of a highly degenerated ZW sex chromosome in the fish Megaleporinus macrocephalus**

**Version: Original Submission** **Date:** 3/28/2024

**Reviewer name: Yusuke Takehana**

**Reviewer Comments to Author:**

The authors assembled a chromosome-level genomic sequence and identified the sex chromosomes of the fish Megaleporinus macrocephalus. This manuscript is potentially interesting because evolution of sex chromosomes and sex-determining genes are one of the most fundamental and popular topics in the evolutionary biology. However, the conceptual advance and the novelty of this study are quite limited. It is another paper adding now one more species to the list of assembled genomes in this fish family. In addition, there is nothing new about the description of the sex chromosomes such as their degenerative signature. Such studies have already been conducted many times and similar conclusions have been reported. Furthermore, the experimental evidence presented appears rather preliminary and is not sufficient to support the claims and interpretations presented in discussion. I am therefore afraid that I have to say that the manuscript does not provide new insights into evolution of sex chromosomes, and thus will not be of sufficient interest to the readers of Gigascience.1. Overall, the paper was very difficult to read due to a lack of logic structure and many errors, such as confusing between males and females, between chromosomes and linkage groups, and so on.2. The introduction is not logically written. It is unclear what is known and to what extent, and why the genome of this species is being determined.3. I did not understand why the authors concluded that Chr13 is the W chromosome and not the Z chromosome. They should assemble the Z and W chromosomes separately and confirm them from different information. It is also unclear how they rule out the possibility that the sequences are chimeric. If they really want to reveal the evolutionary process of sex chromosomes, they should use all the data (Hi-C, linkage analysis, Pool-seq, gene information) to compare the structure of Z and W in detail, including synteny with closely related species.4. The analysis on sex chromosome gene candidates is too poor. Basic analyses have not been conducted on whether these genes are W-specific, whether they are in both Z and W, whether they have paralogs or not on autosomes, how much sequence variation there is, when and in which cells they are expressed, etc.5. All of the discussions are superficial and lacking in logic, and it is unclear what they want to discuss.6. The figures legends are poorly explained, and contain incorrect information, so I don't understand the meaning of the data at all.7. This manuscript contained many grammatical errors leading to many confusing statements, and some sentences that were grammatically correct but awkward meaning. I strongly recommend that the authors seek advice of someone with a good knowledge of English, preferably a native speaker.

**Level of Interest**

Please indicate how interesting you found the manuscript: Choose an item.

**Quality of Written English**

Please indicate the quality of language in the manuscript: Choose an item.

**Declaration of Competing Interests**

Please complete a declaration of competing interests, considering the following questions:

- Have you in the past five years received reimbursements, fees, funding, or salary from an organisation that may in any way gain or lose financially from the publication of this manuscript, either now or in the future?
- Do you hold any stocks or shares in an organisation that may in any way gain or lose financially from the publication of this manuscript, either now or in the future?
- Do you hold or are you currently applying for any patents relating to the content of the manuscript?
- Have you received reimbursements, fees, funding, or salary from an organization that holds or has applied for patents relating to the content of the manuscript?
- Do you have any other financial competing interests?
- Do you have any non-financial competing interests in relation to this paper?

If you can answer no to all of the above, write 'I declare that I have no competing interests' below. If your reply is yes to any, please give details below.

I declare that I have no competing interests' below.

I agree to the open peer review policy of the journal. I understand that my name will be included on my report to the authors and, if the manuscript is accepted for publication, my named report including any attachments I upload will be posted on the website along with the authors' responses. I agree for my report to be made available under an Open Access Creative Commons CC-BY license (http://creativecommons.org/licenses/by/4.0/). I understand that any comments which I do not wish to be included in my named report can be included as confidential comments to the editors, which will not be published.

Choose an item.

To further support our reviewers, we have joined with Publons, where you can gain additional credit to further highlight your hard work (see: https://publons.com/journal/530/gigascience). On publication of this paper, your review will be automatically added to Publons, you can then choose whether or not to claim your Publons credit. I understand this statement.

Yes Choose an item.
