# Supplementary material for: De novo assembly and characterization of a highly degenerated ZW sex chromosome in the fish Megaleporinus macrocephalus
Source: Gigascience. 2024 Nov 26;13:giae085. doi: 10.1093/gigascience/giae085 (PMC11590113; doi:10.1093/gigascience/giae085)
Supplement: giae085_Reviewer_2_Report_Original_Submission — Changwei Shao -- 4/3/2024 [file giae085_reviewer_2_report_original_submission.pdf]

Reviewer Report

Title: De novo assembly and characterization of a highly degenerated ZW sex chromosome in the fish Megaleporinus macrocephalus

Version: Original Submission **Date:** 4/3/2024

Reviewer name: Changwei Shao

Reviewer Comments to Author:

The authors reported the M. macrocephalus reference genome with a highly degenerated ZW sex chromosome and analyzed the expression pattern of sex chromosomes. In a word, this work extends our understanding of the mechanisms of sex chromosome evolution of fish species. The interpretation of the results is sound for the most part, and gives enough proof to verify their results. I just have few concerns as followed.1.On line 54, please confirm it. In the tongue-sole, the size of Z chromosome (21.91Mb) is larger than the W chromosome(16.45Mb).2.On line 88, 89 and 116, the numbers mentioned do not correspond with the results in Figure 1A. Please confirm it.3.In the section on "Gene Prediction and Annotation", a more comprehensive prediction of gene structure can be achieved by combining three methods: de novo prediction, transcriptome prediction, and homology prediction. The results obtained from these three approaches can be integrated using the EVM software, followed by annotation assessment with BUSCO. The method section is somewhat vague and lacks clear logic. For protein prediction, it is advisable to utilize multiple databases, such as SwissProt, InterPro, and Nr, to corroborate evidence from various sources.4.On line 210, there is an error in the caption of Figure 3. Figure 3B should be a colinearity map of the linkage groups and chromosomes.5.The SNP sites identified in females may include those from the Z chromosome, linkage group 23 (LG23) will contain SNP information from both the Z and W chromosomes. This could potentially affect the demarcation of the region of sex conflict.6.On the sex chromosomes, are there candidate genes related to sex differentiation in regions with a high enrichment of specific SNPs? please provide a detailed explanation.7.What is the distribution of genes in the Z and W chromosome-specific regions, and what is the gene loss rate?

Level of Interest

Please indicate how interesting you found the manuscript: Choose an item.

Quality of Written English

Please indicate the quality of language in the manuscript: Choose an item.

Declaration of Competing Interests

Please complete a declaration of competing interests, considering the following questions:

- Have you in the past five years received reimbursements, fees, funding, or salary from an organisation that may in any way gain or lose financially from the publication of this manuscript, either now or in the future?
- Do you hold any stocks or shares in an organisation that may in any way gain or lose financially from the publication of this manuscript, either now or in the future?
- Do you hold or are you currently applying for any patents relating to the content of the manuscript?
- Have you received reimbursements, fees, funding, or salary from an organization that holds or has applied for patents relating to the content of the manuscript?
- Do you have any other financial competing interests?
- Do you have any non-financial competing interests in relation to this paper?

If you can answer no to all of the above, write 'I declare that I have no competing interests' below. If your reply is yes to any, please give details below.

I declare that I have no competing interests

I agree to the open peer review policy of the journal. I understand that my name will be included on my report to the authors and, if the manuscript is accepted for publication, my named report including any attachments I upload will be posted on the website along with the authors' responses. I agree for my report to be made available under an Open Access Creative Commons CC-BY license (http://creativecommons.org/licenses/by/4.0/). I understand that any comments which I do not wish to be included in my named report can be included as confidential comments to the editors, which will not be published.

Choose an item.

To further support our reviewers, we have joined with Publons, where you can gain additional credit to further highlight your hard work (see: https://publons.com/journal/530/gigascience). On publication of this paper, your review will be automatically added to Publons, you can then choose whether or not to claim your Publons credit. I understand this statement.

Yes Choose an item.
