# Supplementary material for: De novo assembly and characterization of a highly degenerated ZW sex chromosome in the fish Megaleporinus macrocephalus
Source: Gigascience. 2024 Nov 26;13:giae085. doi: 10.1093/gigascience/giae085 (PMC11590113; doi:10.1093/gigascience/giae085)
Supplement: giae085_Reviewer_2_Report_Revision_1 — Changwei Shao -- 8/20/2024 [file giae085_reviewer_2_report_revision_1.pdf]

Reviewer Report

Title: De novo assembly and characterization of a highly degenerated ZW sex chromosome in the fish Megaleporinus macrocephalus

Version: Revision 1 **Date:** 8/20/2024

Reviewer name: Changwei Shao

Reviewer Comments to Author:

This study assembled the genome of Megaleporinus macrocephalus, identified the sex chromosomes and the boundaries of pseudoautosomal region and Z/W-specific region, and statistically analyzed the distribution of genes that are differentially expressed in male and female gonads in sex chromosome. It provides a theoretical foundation for the study of heteromorphic sex chromosomes in fish. I suggest making few revisions to the article as a whole.1."These species were formerly classified under the genus Leporinus but were reclassified due to the presence of a unique ZZ/ZW sex chromosome system that emerged at least 12 million years ago" Give examples of species with a ZZ/ZW sex chromosome system and explain whether they have homomorphic or heteromorphic sex chromosomes.2."it remains the only well-characterized W chromosome in fish" It is not only. The homomorphic Z/W chromosomes of Verasper variegatus has published.3.Does M. macrocephalus exhibit sexual dimorphism, and what economic value does this study foresee for it?4."The unique molecular yield of PacBio reads was 56 Gb, and the subread N50 length was 32 kb." The first half of the sentence refers to the number of reads, right? If so, the unit "Gb" is not appropriate to use.5.The content from lines 77 to 80 can be moved to the Methods section.6.The content from lines 95 to 102 is too verbose. It could serve as a caption for Figure 1B.7."as both LG24 and LG27 correspond to different regions of the same sex chromosome." However, as can be seen from Figure 3B, part of LG24 also shows collinearity with some regions of chromosomes 25 and 27. This does not adequately prove that LG24 is part of the sex chromosome; it also includes parts of the autosomes.8."which is undergoing degeneration." What results could corroborate this conclusion?9.Based on information such as linkage groups and defined regions, attempt to assemble the Z and W chromosomes.

Level of Interest

Please indicate how interesting you found the manuscript: Choose an item.

Quality of Written English

Please indicate the quality of language in the manuscript: Choose an item.

Declaration of Competing Interests

Please complete a declaration of competing interests, considering the following questions:

- Have you in the past five years received reimbursements, fees, funding, or salary from an organisation that may in any way gain or lose financially from the publication of this manuscript, either now or in the future?
- Do you hold any stocks or shares in an organisation that may in any way gain or lose financially from the publication of this manuscript, either now or in the future?
- Do you hold or are you currently applying for any patents relating to the content of the manuscript?
- Have you received reimbursements, fees, funding, or salary from an organization that holds or has applied for patents relating to the content of the manuscript?
- Do you have any other financial competing interests?
- Do you have any non-financial competing interests in relation to this paper?

If you can answer no to all of the above, write 'I declare that I have no competing interests' below. If your reply is yes to any, please give details below.

I declare that I have no competing interests

I agree to the open peer review policy of the journal. I understand that my name will be included on my report to the authors and, if the manuscript is accepted for publication, my named report including any attachments I upload will be posted on the website along with the authors' responses. I agree for my report to be made available under an Open Access Creative Commons CC-BY license (http://creativecommons.org/licenses/by/4.0/). I understand that any comments which I do not wish to be included in my named report can be included as confidential comments to the editors, which will not be published.

Choose an item.

To further support our reviewers, we have joined with Publons, where you can gain additional credit to further highlight your hard work (see: https://publons.com/journal/530/gigascience). On publication of this paper, your review will be automatically added to Publons, you can then choose whether or not to claim your Publons credit. I understand this statement.

Yes Choose an item.
